# Efficacy and Safety of Chinese Herbal Medicine for Primary Intracerebral Hemorrhage: A Systematic Review of Randomized Controlled Trials

**DOI:** 10.3389/fphar.2019.01139

**Published:** 2019-10-10

**Authors:** Hui-Lin Wang, Hua Zeng, Meng-Bei Xu, Xiao-Li Zhou, Pei-Qing Rong, Ting-Yu Jin, Qi Wang, Guo-Qing Zheng

**Affiliations:** ^1^Department of Neurology, The Second Affiliated Hospital and Yuying Children’s Hospital of Wenzhou Medical University, Wenzhou, China; ^2^Institute of Clinical Pharmacology, Guangzhou University of Chinese Medicine, Guangzhou, China

**Keywords:** primary intracerebral hemorrhage, Chinese herbal medicine, systematic review, meta-analysis, randomized controlled trials

## Abstract

**Background:** Primary intracerebral hemorrhage (ICH) is the most harmful subtype of stroke, but there have yet been no specific proven therapies. Chinese herbal medicine (CHM) has been used for ICH for more than a thousand years; however, currently it is still lacking of available evidence. The objective of this study is to assess the current available evidence of CHM for acute ICH according to randomized controlled trials.

**Methods:** Eight databases were searched from the year of their respective inception to November 2017. Only the studies that assessed at least four domains with “yes” according to the Cochrane risk of bias tool were selected for analysis. All the data were analyzed by using Review Manager 5.3 software. P < 0.05 was considered to be statistically significant.

**Results:** Forty-five studies with 4,517 individuals were identified. CHM paratherapy can improve dependency, neurological function deficit, volume of hematoma, clinical effective rate, and volume of perihematomal edema compared with CHM alone or placebo (all P < 0.05). By contrast, it was not significant for improving the mortality rate of ICH patients (P > 0.05). In addition, adverse events were reported in 16 studies, whereas 29 studies did not mention it. The frequency of adverse events was 70/972 in the trial group and 48/944 in the control group.

**Conclusion:** The present study provided supportive evidence of CHM for improving dependency of ICH and showed generally safety; however, there is still lack of evidence for improving mortality rate, and it opens for further study.

## Introduction

Primary intracerebral hemorrhage (ICH), known as the irruption of blood in the brain parenchyma, is the most harmful subtype of stroke. It accounts for 10–15% of all strokes and is a devastating stroke with a higher mortality and disability rate compared to ischemic stroke (Liu et al., 2012). There is approximately 40% of case fatality at 1 month with an increase of 54% at 1 year (van Asch et al., 2010; Poon et al., 2014). However, currently no clinically proven specific therapy or treatment is available (Morgenstern et al., 2015). The currently available evidences for the management of ICH recommended by the guidelines remain multifaceted and symptomatic (Steiner et al., 2014; Morgenstern et al., 2015).

Traditional Chinese medicine (TCM) that includes Chinese herbal medicine (CHM), acupuncture, and other non-drug therapies has been used for stroke for thousands of years, but it was difficult to differentiate the ischemic and hemorrhagic stroke. As for Western medicine, during the first half of the 20th century, clinicopathological studies have been concentrated on the clinical symptoms of patients who died after stoke. In 1935, cerebrovascular diseases began to be classified into thrombosis and hemorrhage (intracerebral or subarachnoid) by analyzing stroke patients studied clinically and at necropsy (Caplan, 2011). By the end of the 1980s and the 1990s, computed tomography (CT) and magnetic resonance imaging (MRI) scans became generally available so that brain hemorrhages were readily diagnosed by imaging. Patients could be recognized of accurate classification and differential diagnosis of brain hemorrhages or infarcts when they were alive through CT or MRI. With the introduction of modern Western medicine into China during the Early Republic of China (1912–1949), some doctors recognized that stroke can be either hemorrhagic or ischemic (Zheng and Huang, 2005). In 1978, the Chinese Medical Association approved the classification of cerebrovascular diseases on the Second National Conference on Neuropsychiatry, which clearly divided the stroke into hemorrhagic and ischemic (The Second National Conference on Neuropsychiatry, 1978). Since then, it became the most widely used classification method of cerebrovascular diseases in China. In 1997, stroke in TCM has its national classification standard that exactly same as western medicine. The *National standard of TCM*, section of *Clinical terminology of diagnosis and treatment on TCM* clearly divided it into ischemic and hemorrhagic stroke (China State Bureau of Quality and Technical Supervision, 1997; Zheng and Huang, 2005). Actually, TCM has been the dominantly medical modalities in China before Western medical healthcare was introduced into the regions (Zheng, 2009). It was also the only available medical care of stroke patients in ancient time and now still plays an important role in China and elsewhere worldwide. With the deepening of basic and clinical studies on stroke, TCM has shown its unique academic advantages in the prevention and treatment of stroke, and has gradually exerted a certain influence in the world (Wang, 1997; Wu et al., 2007). At present, there are great differences in the treatment of stroke between China and the West, the most remarkable difference is the use of TCM (Sze et al., 2005). CHM is the main pharmacological therapeutic method in TCM. In addition, systematic review of the rigorous randomized controlled trials (RCTs) has been recognized the highest level of evidence (Parnianfard et al., 2017). The validity of a conclusion of systematic review is highly dependent on the quality of RCTs included. Conversely, the low-quality RCTs with high risk of bias and high heterogenicity have the positive conclusions, which are not scientifically sound and misleading attribute to methodological flaws (Wang et al., 2019). Correspondingly, the Cochrane group guidelines for clinical reviews have developed a strict process to exclude such not-so-good studies when conducted a systematic review (Chan et al., 2012). Thus, the objective of the present systematic review is to assess the existing evidence of CHM for ICH after the exclusion of not-so-good RCTs.

## Methods

Ethical approval was not needed because of literature research. The systematic review was conducted according to the Preferred Reporting Items for Systematic Reviews and Meta-analyses: The PRISMA Statement (Moher et al., 2009).

### Database and Search Strategies

Electronic searches were performed in eight databases from their respective inception to November 2017: PubMed, EMBASE, Web of Science, Cochrane Library, Chinese National Knowledge Infrastructure, Chinese Biomedical Literature Database, Chinese VIP Database, and Wanfang Database. We also manually searched the additional relevant studies using the references of the systematic reviews published previously. No language restrictions were applied. The following search strategy was used for PubMed and was modified to suit other databases.

1. Chinese medicine*
2. Chinese herbal*
3. integrative medicine
4. OR/1–3
5. intracerebral hemorrhage*
6. hemorrhagic stroke*
7. OR/4–5
8. 4 AND 6

### Eligibility Criteria

#### Types of Studies

Only RCTs that assessed the efficacy and safety of CHM for acute ICH were included, regardless of publication status or language. If the study had a three-arm design, we extracted data only for the group(s) involving CHM and the control group(s). Quasi-randomized trials, such as those in which patients were allocated according to date of birth and order of admission number, were excluded.

#### Types of Participants

We included participants with a diagnosis of ICH within 7 days of stroke onset in accordance with the diagnostic criteria of Chinese Cerebrovascular Disease Diagnosis Standard (CCDDS) made by the Chinese Medical Association at the Fourth National Conference on cerebrovascular disease in 1995 (The Fourth National Conference on Cerebrovascular Disease, 1996), CCDDS in 1998 (Wang, 1998), and guidelines for prevention and treatment of cerebrovascular diseases issued by the *Neurology Branch of Chinese Medical* Association in 2007 (Rao, 2007), regardless of gender, age, or race. All participants were confirmed with CT/MRI scan.

#### Types of Interventions

The analyzed intervention was CHM adjunct western conventional medication (WCM), regardless of dosage, duration, administrated methods, administration route, or administration time of treatment. The comparator was given WCM alone or plus CHM placebo. WCM refers to the combination of needed therapies of the following aspects (Morgenstern et al., 2015): 1) general supportive care; 2) blood pressure management; 3) glucose management; 4) hemostasis and coagulopathy; patients with a severe coagulation factor deficiency or severe thrombocytopenia should receive appropriate factor replacement therapy or platelets, respectively; 5) surgical treatment; 6) management of medical complications; and 7) rehabilitation and recovery. Studies comparing one kind of CHM with another CHM were excluded.

#### Types of Outcomes

Mortality and dependency as primary outcomes were measured at the end of the treatment course and the follow-up period. Dependency has the definition of needing the aid of activity of daily living (ADL), which was measured by a standard rating scale such as the Barthel Index (BI), modified Rankin Standard (mRS), ADL Scale, Glasgow Outcome Scale (GOS), and the degree of disability. The degree of disability was recorded in the standard of the degree of clinical neurological deficit in stroke patients (SDCNFS1995) (Chen, 1996). The secondary outcomes were the neurological deficit improvement (Li et al., 2015), volume of hematoma (VH), the clinical effective rate, volume of perihematomal edema (VPE), and adverse events. The neurological deficit improvement was measured after treatment using National Institutes of Health Stroke Scale (NIHSS) score and Chinese Clinical Neurological Deficit Scale (CCNDS). A serious adverse event (SAE) is defined as “any untoward medical occurrence at any dose”: a) results in death; b) is life-threatening; c) requires inpatient hospitalization or prolongation of an existing hospitalization; d) results in persistent or significant disability/incapacity; or e) is a congenital anomaly/birth defect (ICH Expert Working Group, 1996).

### Data Extraction and Management

We extracted data that included first author’s name; publication year; diagnosis standard of ICH; study design; patients’ total number and characteristics (age, gender, belong to control or treatment group); inclusion of patient-based VH; intervention schedule and intervention time for treatment group and control group; follow-up time; change of outcome index; the reports of adverse reaction. The outcome information of the last evaluation would be extracted if there were multiple time-point outcome indicators. Reasons for the exclusion of studies were also recorded. For eligible studies, two authors extracted data independently and resolved all discrepancies by discussing with each other or with a third author.

### Assessment of Risk of Bias

The Cochrane’s Collaboration tool was used to assess the risk of bias by the 7-item criteria (Higgins et al., 2008). Two authors valued the eligible studies independently and a discussion with the corresponding author was conducted to solve any discrepancies. RCTs that received at least four out of seven domains “yes” were selected for analysis (Li et al., 2015; Yang et al., 2017)

### CHM Composition

The main compositions of the CHM formulae were recorded. We calculated the frequency of use of all Chinese herbs, and analyzed and described in detail those used at high frequency.

### The Quality of the Clinical Studies

In order to assess the quality of the clinical studies, we used a rating system (Wang et al., 2019) as follows: 1) high quality—full information about the botanical material is provided, including a voucher specimen; 2) moderate quality—only partial information about the botanical material is provided and a voucher specimen is missed; there are taxonomic inaccuracies; 3) low quality—inadequate information and overall taxonomically inadequate.

### Statistical Analysis

Data were analyzed by using Review Manager (version 5.3). P < 0.05 was considered statistically significant. Dichotomous outcomes were calculated by the risk ratio (RR), with a 95% confidence interval (CI), whereas continuous outcomes were calculated by weighted mean differences (WMD) or standardized mean differences (SMD). The Cochrane Q-statistic test and the I^2^-statistic were used to test the heterogeneity among studies. When no obvious heterogeneity exists (P > 0.1, I^2^ < 50%), we used a fixed effect model. Otherwise, the random effect model is a more plausible match. However, all meta-analyses were carried out through a random-effect model because of the clinical heterogeneity. Publication bias was visually estimated using funnel plots.

## Results

### Description of Studies

**Figure 1:**
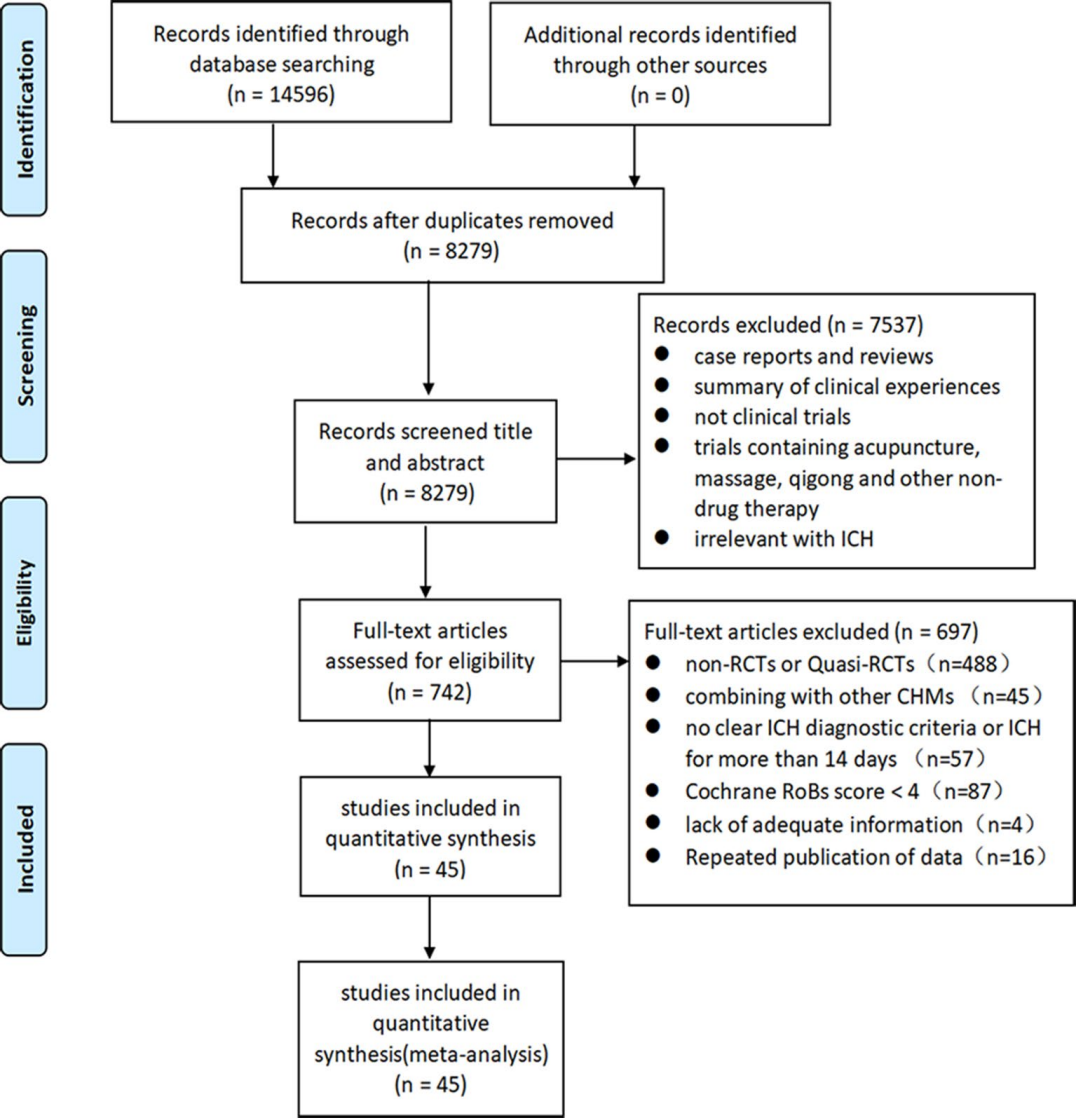
Flowchart of study screening.

We identified 14,596 potentially relevant hints from eight databases. Removing duplication of literature, there were 8,279 articles left. We excluded 7,514 studies that are not related to this study after reading the titles and abstracts in detail. Through reading the full text of 742 articles, 697 articles were excluded for at least one of the following reasons: 1) non-randomized or quasi-RCTs; 2) no clear ICH diagnostic criteria; 3) combined Chinese herbs with other TCM treatment modalities; 4) published by repeated data; and 5) unavailable data. Finally, 45 articles were included for analysis (**Figure 1**).

### Characteristics of Included Studies

The sample sizes of the 45 studies ranged from 51 to 404. All studies were published between 2000 and 2017. There were 4,517 participants of Chinese ethnicity that were included in the 45 studies, of which 2,267 patients were treatment groups and 2,250 patients served as controls. Forty-two studies (Fan et al., 2000; Jia et al., 2000; Dai et al., 2002; Ma and Gong, 2005; Yang and Liu, 2006; Fan et al., 2008; Sun and Chen, 2008; Huang et al., 2010; Liao et al., 2010; Ming et al., 2010; Chen, 2011; Li et al., 2011; Peng et al., 2011; Wang and Wang, 2011; Li et al., 2012; Shen et al., 2012; Zhang et al., 2012; Ming et al., 2013; Wang et al., 2013; Bi and Hu, 2014; Gu et al., 2014; Guo et al., 2014; Li, 2014; Ye, 2014; Guan et al., 2015; Li et al., 2015; Luo et al., 2015; Peng and Feng, 2015; Shen and Luo, 2015; Guo et al., 2016; Jiang et al., 2016; Li et al., 2016; Liu and Wu, 2016; Liu and Zhang, 2016; Long et al., 2016; Shang and Yang, 2016; Xia et al., 2016; Zhou et al., 2016; Lei and Qiao, 2017; Ma and Zhang, 2017; Sun et al., 2017; Zhang et al., 2017) compared CHM plus WCM with WCM alone, and three studies (Huang et al., 2006; Chen et al., 2010; Liu et al., 2012) compared CHM plus WCM with CHM placebo plus WCM. The CHM treatment course varied from 7 to 90 days. Nineteen studies reported follow-up from 7 days to 1 year. The follow-up time over 3 months was reported in 18 studies (Jia et al., 2000; Dai et al., 2002; Huang et al., 2006; Chen et al., 2010; Huang et al., 2010; Wang and Wang, 2011; Li et al., 2012; Shen et al., 2012; Zhang et al., 2012; Ming et al., 2013; Wang et al., 2013; Ye, 2014; Li et al., 2015; Li and Wu, 2016; Liu and Zhang, 2016; Xia et al., 2016; Ma and Zhang, 2017). Mortality rate was observed in 14 studies; dependence in 10 studies; clinical effective rate in 28 studies; neurological deficit score in 37 studies; VH in 23 studies; VPE in 7 studies; and adverse events in 16 studies. The detailed characteristics are listed in **Supplementary Table 1**.

### Risk of Bias in Included Studies

The total scores according to Cochrane risk of bias (**Table 1**) are as follows: Xia et al. (2016) with 7 points; Chen et al. (2010) and Li et al.(2016) with 6 points; Huang et al. (2006) and Luo et al. (2015) with 5 points; the other 40 RCTs with 4 points. The method of random distribution was clearly proposed in all included documents. In addition, three studies (Chen et al., 2010; Li et al., 2016; Xia et al., 2016) explicitly proposed the method of distribution concealment; two studies (Chen et al., 2010; Xia et al., 2016) had double blinding for the implementers and participants; and four studies (Huang et al., 2006; Luo et al., 2015; Li et al., 2016; Xia et al., 2016) evaluated the outcome indexes by blind method.

**Table 1:** Assessment of study quality and risk of bias.

First author, year	7-item criteria							
	A	B	C	D	E	F	G	Total
Fan et al., 2000	+	–	–	–	+	+	+	4
Jia et al., 2000	+	–	–	–	+	+	+	4
Dai et al., 2002	+	–	–	–	+	+	+	4
Ma and Gong, 2005	+	–	–	–	+	+	+	4
Huang et al., 2006	+	–	–	+	+	+	+	5
Yang and Liu, 2006	+	–	–	–	+	+	+	4
Fan et al., 2008	+	–	–	–	+	+	+	4
Sun and Chen, 2008	+	–	–	–	+	+	+	4
Liao et al., 2010	+	–	–	–	+	+	+	4
Chen et al., 2010	+	+	+	–	+	+	+	6
Huang et al., 2010	+	–	–	–	+	+	+	4
Ming et al., 2010	+	–	–	–	+	+	+	4
Chen, 2011	+	–	–	–	+	+	+	4
Li et al., 2011	+	–	–	–	+	+	+	4
Peng et al., 2011	+	–	–	–	+	+	+	4
Wang and Wang, 2011	+	–	–	–	+	+	+	4
Li et al., 2012	+	–	–	–	+	+	+	4
Liu et al., 2012	+	–	–	–	+	+	+	4
Shen et al., 2012	+	–	–	–	+	+	+	4
Zhang et al., 2012	+	–	–	–	+	+	+	4
Ming et al., 2013	+	–	–	–	+	+	+	4
Wang et al., 2013	+	–	–	–	+	+	+	4
Bi and Hu, 2014	+	–	–	–	+	+	+	4
Gu et al., 2014	+	–	–	–	+	+	+	4
Guo et al., 2014	+	–	–	–	+	+	+	4
Li, 2014	+	–	–	–	+	+	+	4
Ye, 2014	+	–	–	–	+	+	+	4
Guan et al., 2015	+	–	–	–	+	+	+	4
Li et al., 2015	+	–	–	–	+	+	+	4
Luo et al., 2015	+	–	–	+	+	+	+	5
Peng and Feng, 2015	+	–	–	–	+	+	+	4
Shen and Luo, 2015	+	–	–	–	+	+	+	4
Li et al., 2016	+	+	–	+	+	+	+	6
Guo et al., 2016	+	–	–	–	+	+	+	4
Jiang et al., 2016	+	–	–	–	+	+	+	4
Liu and Wu, 2016	+	–	–	–	+	+	+	4
Liu and Zhang, 2016	+	–	–	–	+	+	+	4
Long et al., 2016	+	–	–	–	+	+	+	4
Shang and Yang, 2016	+	–	–	–	+	+	+	4
Xia et al., 2016	+	+	+	+	+	+	+	7
Zhou et al., 2016	+	–	–	–	+	+	+	4
Lei and Qiao, 2017	+	–	–	–	+	+	+	4
Ma and Zhang, 2017	+	–	–	–	+	+	+	4
Sun et al., 2017	+	–	–	–	+	+	+	4
Zhang et al., 2017	+	–	–	–	+	+	+	4

A to G, the 7-Item criteria. A, adequate sequence generation; B, concealment of allocation; C, blinding of participants and personnel; D, blinding of outcome assessment; E, incomplete out-come data; F, selective reporting; G, other bias; +: low risk of bias, –: high risk of bias.

### The Quality of the Clinical Studies

We accessed the quality of the included clinical studies with a rating system, which is related to the information about the botanical material and voucher specimens. All studies are low quality with inadequate information and overall taxonomically inadequate. The quality for each included clinical study is summarized in **Table 2**.

**Table 2:** The quality of the clinical studies.

First author, year	Botanical material information	Voucher specimen	Quality
Fan et al., 2000	I	−	Low
Jia et al., 2000	I	−	Low
Dai et al., 2002	I	−	Low
Ma and Gong, 2005	I	−	Low
Huang et al., 2006	I	−	Low
Yang and Liu, 2006	I	−	Low
Fan et al., 2008	I	−	Low
Sun and Chen, 2008	I	−	Low
Liao et al., 2010	I	−	Low
Chen et al., 2010	I	−	Low
Huang et al., 2010	I	−	Low
Ming et al., 2010	I	−	Low
Chen, 2011	I	−	Low
Li et al., 2011	I	−	Low
Peng et al., 2011	I	−	Low
Wang and Wang, 2011	I	−	Low
Li et al., 2012	I	−	Low
Liu et al., 2012	I	−	Low
Shen et al., 2012	I	−	Low
Zhang et al., 2012	I	−	Low
Ming et al., 2013	I	−	Low
Wang et al., 2013	I	−	Low
Bi and Hu, 2014	I	−	Low
Gu et al., 2014	I	−	Low
Guo et al., 2014	I	−	Low
Li, 2014	I	−	Low
Ye, 2014	I	−	Low
Guan et al., 2015	I	−	Low
Li et al., 2015	I	−	Low
Luo et al., 2015	I	−	Low
Peng and Feng, 2015	I	−	Low
Shen and Luo, 2015	I	−	Low
Li et al., 2016	I	−	Low
Guo et al., 2016	I	−	Low
Jiang et al., 2016	I	−	Low
Liu and Wu, 2016	I	−	Low
Liu and Zhang, 2016	I	−	Low
Long et al., 2016	I	−	Low
Shang and Yang, 2016	I	−	Low
Xia et al., 2016	I	−	Low
Zhou et al., 2016	I	−	Low
Lei and Qiao, 2017	I	−	Low
Ma and Zhang, 2017	I	−	Low
Sun et al., 2017	I	−	Low
Zhang et al., 2017	I	−	Low

F, Full information about the botanical material is provided; P, Partial information about the botanical material is provided; I, Inadequate information about the botanical material is provided; +, includes a voucher specimen; -, a voucher specimen is missing.

### Effectiveness

#### Mortality

**Figure 2:**
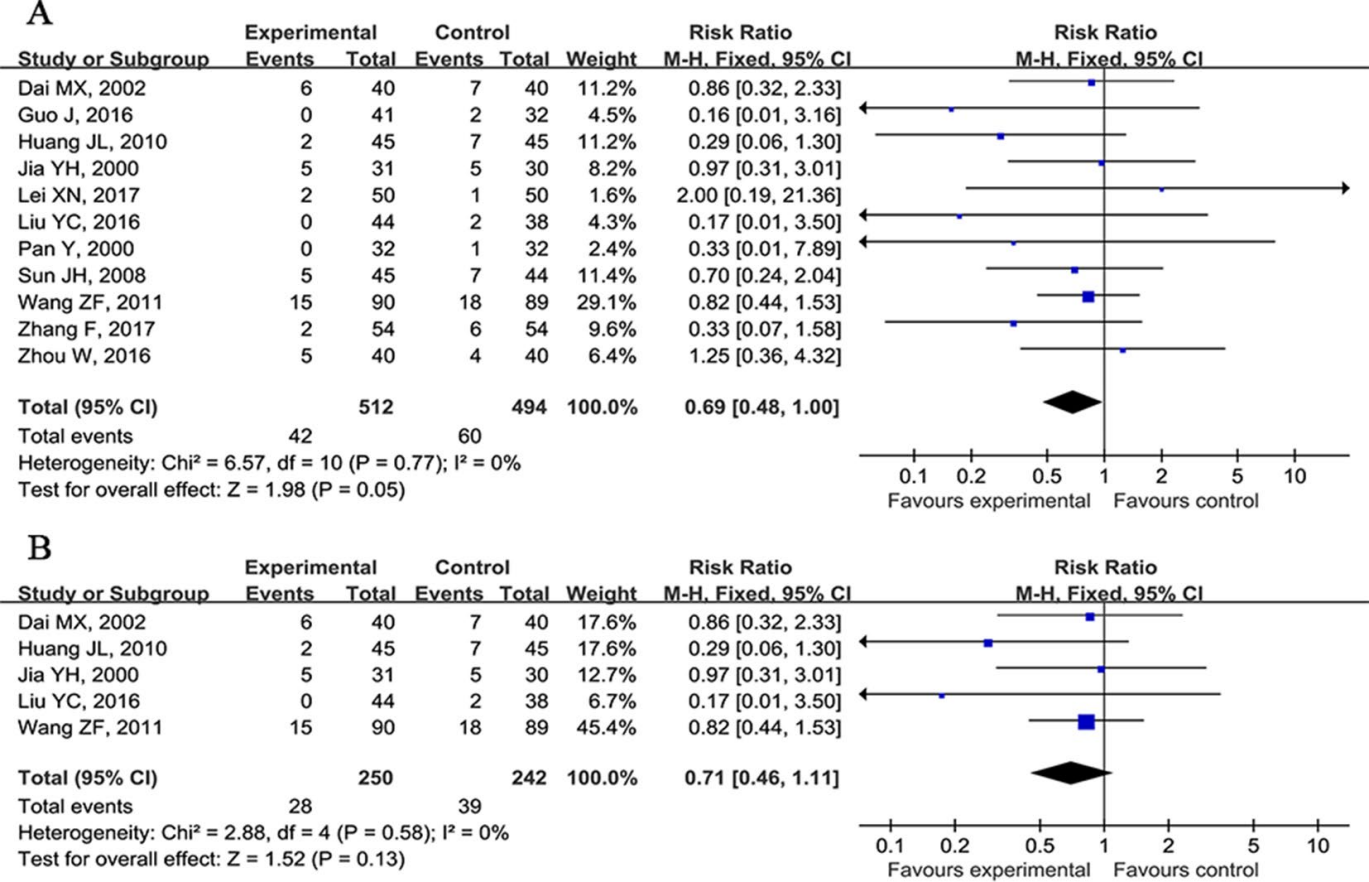
Forest plots of meta-analysis of mortality rate. **(A)** Eleven studies compared CHM plus WCM with WCM alone. **(B)** Five studies that followed up for more than 3 months compared CHM plus WCM versus WCM alone in acute ICH patients.

Fourteen studies (Fan et al., 2000; Jia et al., 2000; Dai et al., 2002; Huang et al., 2006; Sun and Chen, 2008; Chen et al., 2010; Huang et al., 2010; Wang and Wang, 2011; Liu et al., 2012; Guo et al., 2016; Liu and Zhang, 2016; Zhou et al., 2016; Lei and Qiao, 2017; Zhang et al., 2017) reported the mortality rate as outcome measure. Three studies (Huang et al., 2006; Chen et al., 2010; Liu et al., 2012) showed CHM plus WCM was not significant for improving mortality rate compared with CHM placebo plus WCM (p > 0.05). Meta-analysis of 11 studies (Fan et al., 2000; Jia et al., 2000; Dai et al., 2002; Sun and Chen, 2008; Huang et al., 2010; Wang and Wang, 2011; Guo et al., 2016; Liu and Zhang, 2016; Zhou et al., 2016; Lei and Qiao, 2017; Zhang et al., 2017) showed that CHM plus WCM was not significant for improving the mortality rate in acute ICH patients compared with WCM alone (n = 1006, nT/nC = 512/494, RR 0.69, 95%CI: 0.48∼1.00, P = 0.05, heterogeneityχ^2^ = 6.57, df = 10, p = 0.94, I^2^ = 0%) (**Figure 2A**); meta-analysis of five studies (Jia et al., 2000; Dai et al., 2002; Huang et al., 2010; Wang and Wang, 2011; Liu and Zhang, 2016) that followed up for more than 3 months showed similar results (n = 492, nT/nC = 250/242, RR 0.71, 95% CI: 0.46∼1.11, P = 0.13, heterogeneity χ^2^ = 2.88, df = 4, p = 0.58, I^2^ = 0%) (**Figure 2B**).

#### Dependency

**Figure 3:**
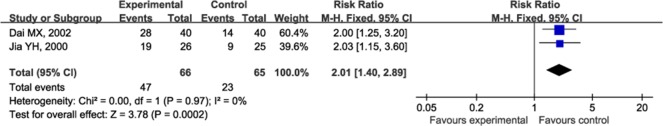
Forest plot of meta-analysis of acute ICH patients’ dependency (≥3 months follow-up) among two studies compared CHM plus WCM with WCM alone.

Ten studies (Jia et al., 2000; Dai et al., 2002; Huang et al., 2006; Chen et al., 2010; Wang and Wang, 2011; Li et al., 2012; Liu et al., 2012; Li et al., 2016; Xia et al., 2016; Ma and Zhang, 2017) used the dependency as outcome measure. Three studies showed that CHM plus WCM was signiﬁcant for reducing the dependence according to BI (Huang et al., 2006); combined use of mRS and BI (Chen et al., 2010) and home-made comparable scale (Liu et al., 2012) compared with CHM placebo plus WCM (P < 0.05). Seven studies (Jia et al., 2000; Dai et al., 2002; Wang and Wang, 2011; Li et al., 2012; Li et al., 2016; Xia et al., 2016; Ma and Zhang, 2017) compared the CHM plus WCM with WCM alone, and all showed that CHM plus WCM was significant for improving dependency at the end of the treatment and follow-up more than 3 months according to BI (Li et al., 2012), mRS (Li et al., 2016; Xia et al., 2016), GOS (Ma and Zhang, 2017), ADL (Wang and Wang, 2011), and degree of disability (Jia et al., 2000; Dai et al., 2002) (P < 0.05). Meta-analysis of two studies (Jia et al., 2000; Dai et al., 2002) showed CHM plus WCM was significant for improving degree of disability (n = 131, nT/nC = 66/65, RR 2.01, 95% CI: 1.40∼2.89, P < 0.0001, heterogeneity χ^2^ = 0.00, df = 1, p = 0.97, I^2^ = 0%) at the end of the treatment compared with WCM (**Figure 3**).

#### Clinical Effective Rate

**Figure 4:**
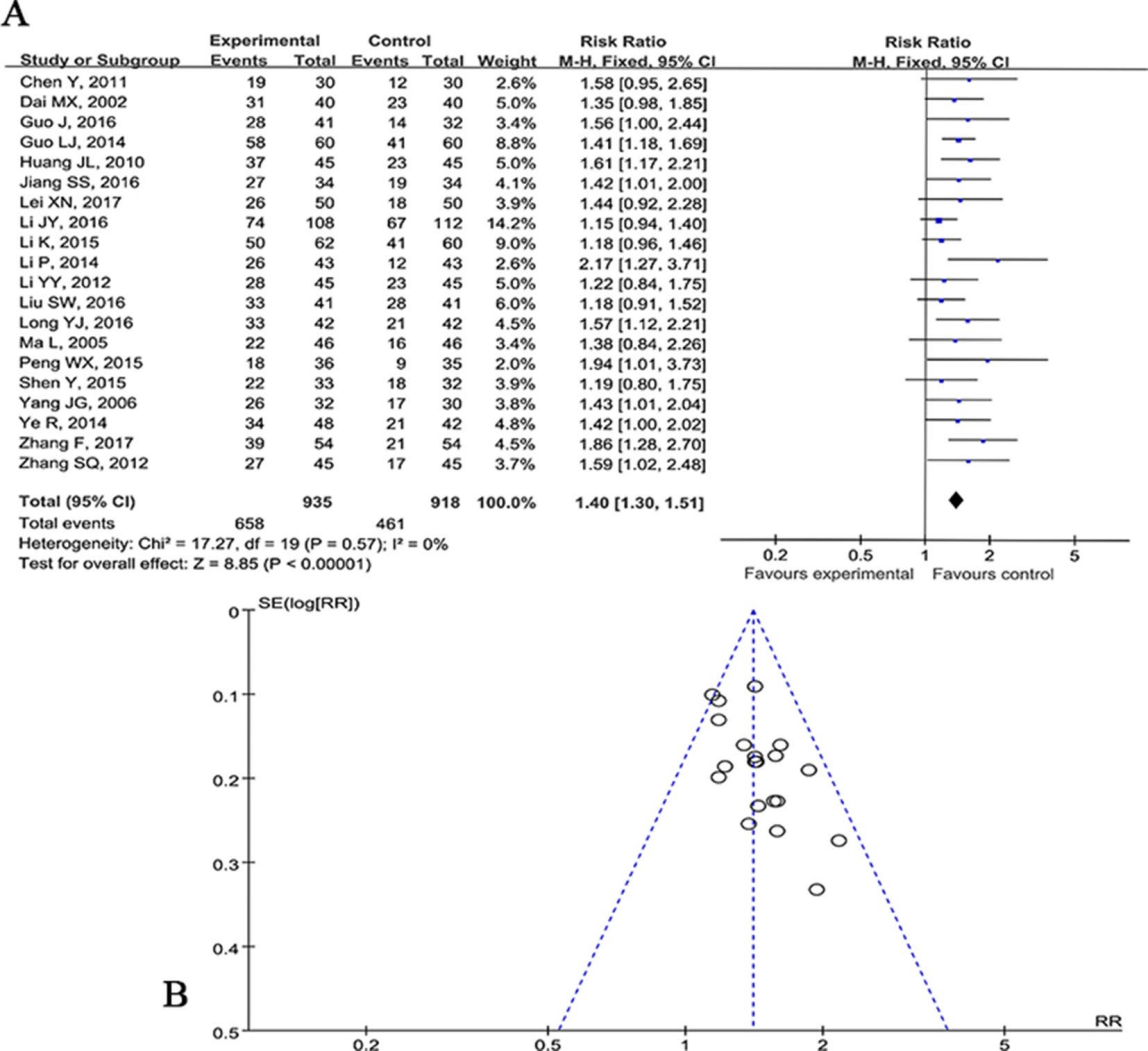
**(A)** Forest plot of meta-analysis of clinical effective rate among 20 studies compared CHM plus WCM versus WCM alone. **(B)** Funnel graph of publication bias.

The clinical efficacy was reported in 28 studies (Fan et al., 2000; Jia et al., 2000; Dai et al., 2002; Ma and Gong, 2005; Huang et al., 2006; Yang and Liu, 2006; Huang et al., 2010; Chen, 2011; Li et al., 2012; Liu et al., 2012; Zhang et al., 2012; Guo et al., 2014; Li, 2014; Ye, 2014; Guan et al., 2015; Li et al., 2015; Peng and Feng, 2015; Shen and Luo, 2015; Guo et al., 2016; Jiang et al., 2016; Li et al., 2016; Liu and Wu, 2016; Long et al., 2016; Shang and Yang, 2016; Zhou et al., 2016; Lei and Qiao, 2017; Sun et al., 2017; Zhang et al., 2017). Meta-analysis of two studies (Huang et al., 2006; Liu et al., 2012) showed CHM plus WCM was significant for increasing clinical effective rate compared with CHM placebo plus WCM (P < 0.05). Meta-analysis of 20 studies (Dai et al., 2002; Ma and Gong, 2005; Yang and Liu, 2006; Huang et al., 2010; Chen, 2011; Li et al., 2012; Zhang et al., 2012; Guo et al., 2014; Li, 2014; Ye, 2014; Li et al., 2015; Peng and Feng, 2015; Shen and Luo, 2015; Guo et al., 2016; Jiang et al., 2016; Li et al., 2016; Liu and Wu., 2016; Long et al., 2016; Lei and Qiao, 2017; Zhang et al., 2017) showed CHM plus WCM was significant for increasing clinical effective rate compared with WCM alone according to SDCNFS 1995 (n = 1853, nT/nC = 935/918, RR 1.40, 95%CI: 1.30∼1.51, P < 0.00001, heterogeneity χ^2^ = 17.27, df = 19, p = 0.57, I^2^ = 0%) (**Figure 4A**). The funnel graph is basically symmetrical, indicating that there is no obvious publication bias (**Figure 4B**). Owing to the different evaluation criteria of clinical efficiency, the other six studies (Fan et al., 2000; Jia et al., 2000; Guan et al., 2015; Shang and Yang, 2016; Zhou et al., 2016; Sun et al., 2017) failed to conduct meta-analysis, but they showed positive results (P < 0.05).

#### CCNDS Score

**Figure 5:**
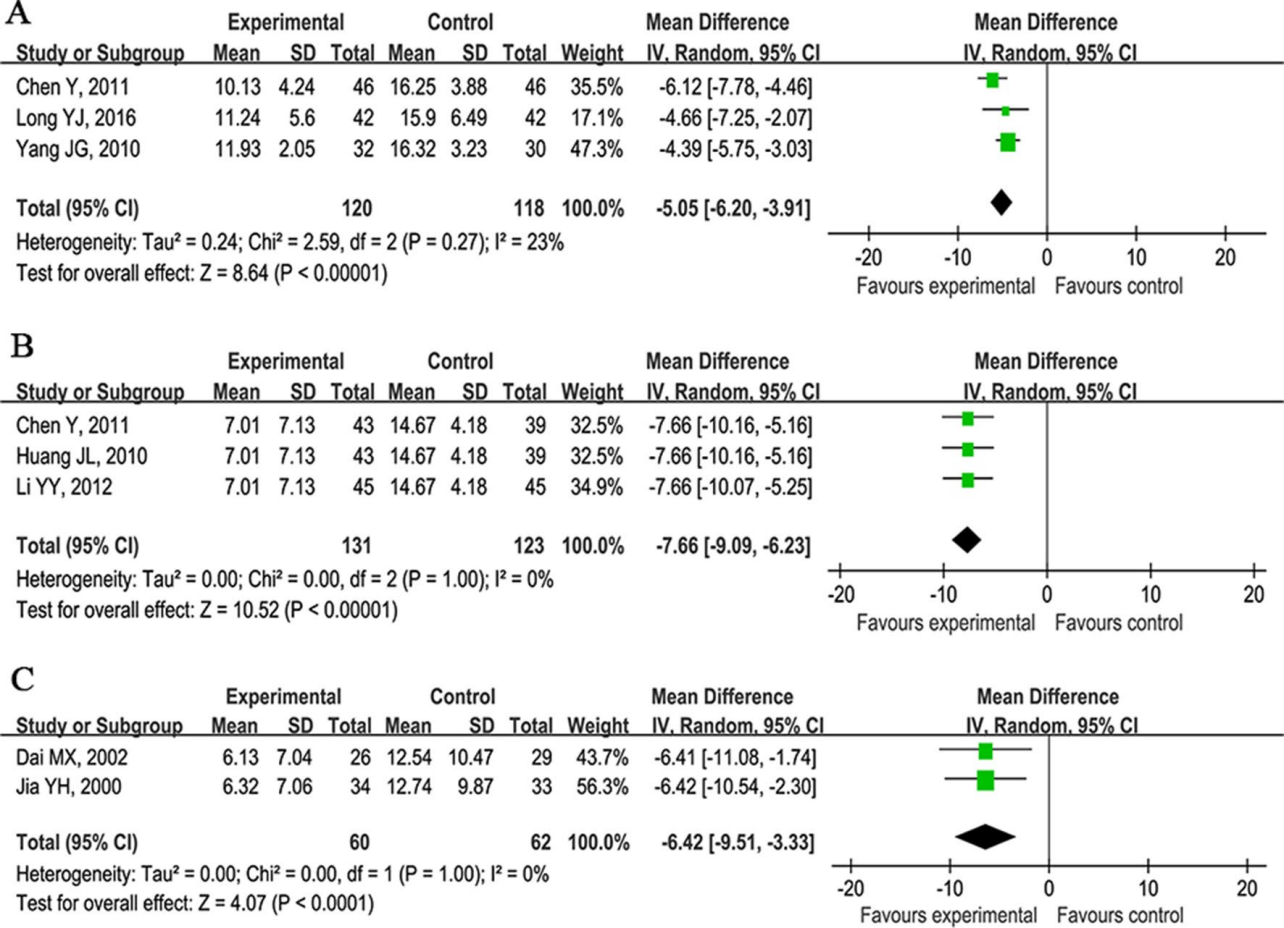
Forest plots of meta-analysis of Chinese clinical neurological deficit scale score. **(A)** Three studies compared CHM plus WCM with WCM alone at 14 days. **(B)** Three studies at 90 days and **(C)** two studies at 180 days.

The CCNDS score as outcome measure was used in 12 studies (Jia et al., 2000; Dai et al., 2002; Ma and Gong, 2005; Yang and Liu, 2006; Huang et al., 2010; Chen, 2011; Wang and Wang, 2011; Li et al., 2012; Liu et al., 2012; Wang et al., 2013; Peng and Feng, 2015; Long et al., 2016). One study (Liu et al., 2012) showed that CHM plus WCM was significant for reducing the CCNDS score at 48 days compared with CHM placebo plus WCM (P < 0.05). Eleven studies compared CHM plus WCM with WCM alone at 14, 90, or 180 days. Meta-analysis of three studies (Ma and Gong, 2005; Yang and Liu, 2006; Long et al., 2016) showed CHM plus WCM was significant for reducing the CCNDS score at 14 days compared with WCM (n = 238, nT/nC = 120/118, WMD −5.05, 95%CI: −6.20∼−3.91, P < 0.00001, heterogeneity χ^2^ = 2.59, df = 2, p = 0.27, I^2^ = 23%; **Figure 5A**); three studies (Huang et al., 2010; Chen, 2011; Li et al., 2012) at 90 days (n = 254, nT/nC = 131/123, WMD −7.66, 95%CI: −9.09∼−6.23, P < 0.00001, heterogeneity χ^2^ = 0.00, df = 2, p = 1.00, I^2^ = 0%; **Figure 5B**); and two studies (Jia et al., 2000; Dai et al., 2002) at 180 days (n = 122, nT/nC = 60/62, WMD −6.42, 95%CI: −9.51∼−3.33, P < 0.00001, heterogeneity χ^2^ = 0.00, df = 1, p = 1.00, I^2^ = 0%; **Figure 5C**). Due to the inconsistent time points of evaluation, the other three studies (Wang and Wang, 2011; Wang et al., 2013; Peng and Feng, 2015) failed to conduct meta-analysis, but they reported positive results.

#### NIHSS Score

Nineteen studies (Huang et al., 2006; Fan et al., 2008; Sun and Chen, 2008; Liao et al., 2010; Shen et al., 2012; Zhang et al., 2012; Gu et al., 2014; Li, 2014; Guan et al., 2015; Luo et al., 2015; Jiang et al., 2016; Li et al., 2016; Liu and Wu, 2016; Liu and Zhang, 2016; Shang and Yang, 2016; Zhou et al., 2016; Lei and Qiao, 2017; Ma and Zhang, 2017; Sun et al., 2017) used NIHSS scores as outcome. Huang et al. (2006) showed that CHM plus WCM was significant for improving the NIHSS score compared with CHM placebo plus WCM at 90 days (P < 0.05). Eighteen studies (Fan et al., 2008; Sun and Chen, 2008; Liao et al., 2010; Shen et al., 2012; Zhang et al., 2012; Gu et al., 2014; Li, 2014; Guan et al., 2015; Luo et al., 2015; Jiang et al., 2016; Li et al., 2016; Liu and Wu, 2016; Liu and Zhang, 2016; Shang and Yang, 2016; Zhou et al., 2016; Lei and Qiao, 2017; Ma and Zhang, 2017; Sun et al., 2017) showed CHM plus WCM exerted a significantly better recovery of lost neurological functions than that of WCM alone (P < 0.05). We failed to conduct meta-analysis because of the various assessment time points from 7 to 90 days after CHM treatment.

#### VH

**Figure 6:**
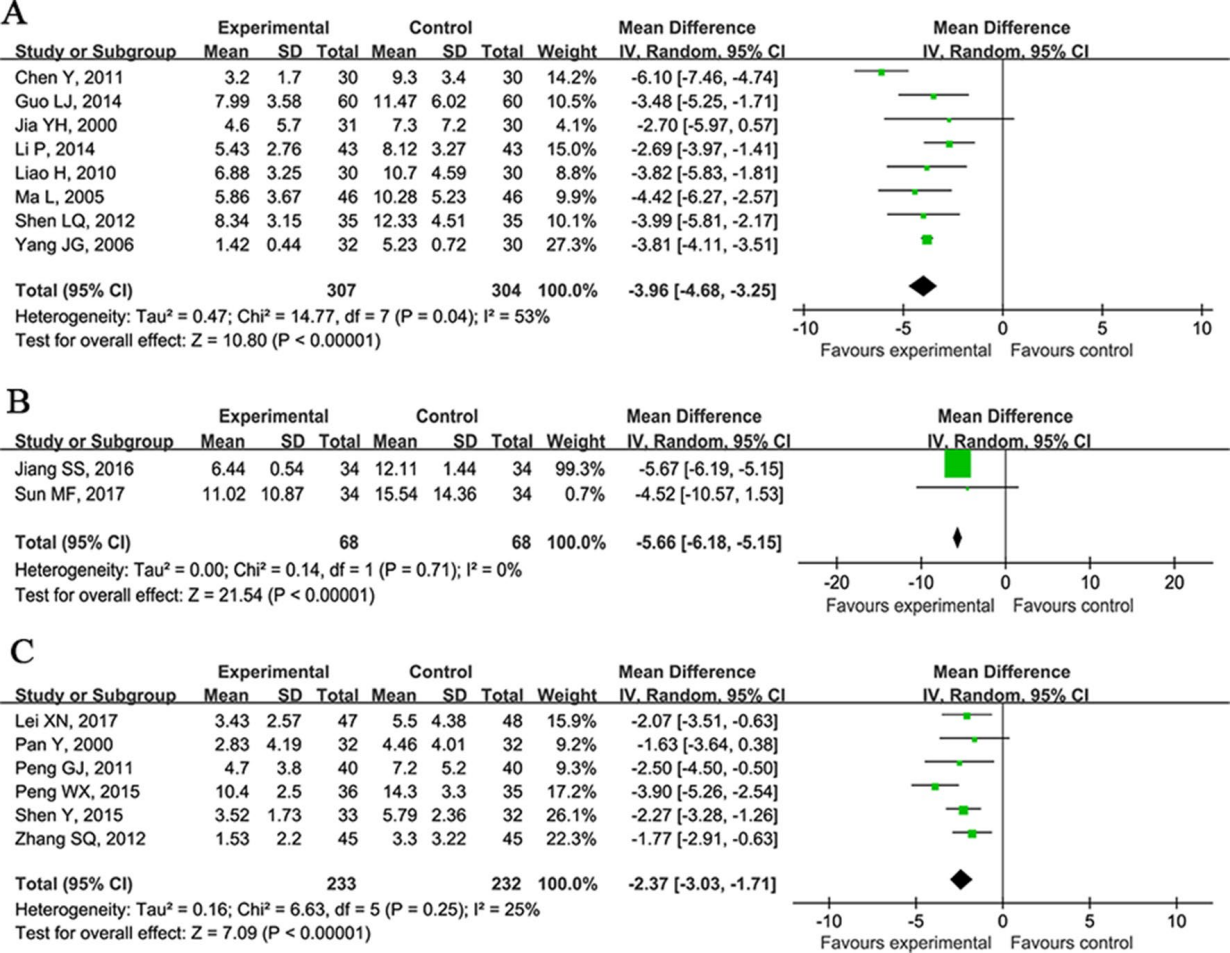
Forest plots of meta-analysis of volume of hematoma. **(A)** Eight studies compared CHM plus WCM with WCM alone at 7 days. **(B)** Two studies at 21 days and **(C)** six studies at 28 days.

It was reported as an outcome measure in 23 studies (Fan et al., 2000; Jia et al., 2000; Ma and Gong, 2005; Yang and Liu, 2006; Liao et al., 2010; Ming et al., 2010; Chen, 2011; Li et al., 2011; Peng et al., 2011; Shen et al., 2012; Zhang et al., 2012; Wang et al., 2013; Bi and Hu, 2014; Guo et al., 2014; Li, 2014; Ye, 2014; Luo et al., 2015; Peng and Feng, 2015; Jiang et al., 2016; Li et al., 2016; Lei and Qiao, 2017; Sun et al., 2017) assessed at 7, 21, and 28 days after CHM treatment. Meta-analysis of eight studies (Jia et al., 2000; Ma and Gong, 2005; Yang and Liu, 2006; Liao et al., 2010; Chen, 2011; Shen et al., 2012; Guo et al., 2014; Li, 2014) showed that CHM plus WCM significantly reduced the VH at 7 days compared with WCM alone (n = 611, nT/nC = 307/304, WMD −3.96, 95%CI: −4.68∼−3.25, P < 0.00001, heterogeneity χ^2^ = 14.77, df = 7, p = 0.04, I^2^ = 53%; **Figure 6A**); two studies (Jiang et al., 2016; Sun et al., 2017) at 21 days (n = 124, nT/nC = 68/68, WMD -5.66, 95%CI: −6.18∼−5.15, P < 0.00001, heterogeneity χ^2^ = 0.14, df = 1, p = 0.71, I^2^ = 0%; **Figure 6B**); and six studies (Fan et al., 2000; Peng et al., 2011; Zhang et al., 2012; Ming et al., 2013; Li, 2014; Ye, 2014; Long et al., 2016) at 28 days (n = 465, nT/nC = 233/232, WMD −2.37, 95%CI: −3.03∼−1.71, P < 0.00001, heterogeneity χ^2^ = 6.63, df = 5, p = 0.25, I^2^ = 25%; **Figure 6C**). Seven studies (Ming et al., 2010; Li et al., 2011; Wang et al., 2013; Bi and Hu, 2014; Ye, 2014; Luo et al., 2015; Li et al., 2016) failed to carry out meta-analysis because of the inconsistent time points of evaluation, but they reported positive results (P < 0.05).

#### VPE

It was reported in 7 studies at 14 days (Li, 2014; Long et al., 2016), 21 days (Sun and Chen, 2008; Ming et al., 2013; Ye, 2014), and 28 days (Peng et al., 2011; Zhang et al., 2012) after CHM treatment. These studies showed CHM plus WCM significantly lessened VPE compared with WCM alone (P < 0.05).

#### Adverse Events

They were reported in 16 studies (Fan et al., 2000; Huang et al., 2006; Chen et al., 2010; Li et al., 2011; Peng et al., 2011; Gu et al., 2014; Li, 2014; Guan et al., 2015; Luo et al., 2015; Peng and Feng, 2015; Li et al., 2016; Liu and Wu, 2016; Xia et al., 2016; Zhou et al., 2016; Lei and Qiao, 2017), whereas 29 studies did not mention it. The frequency of adverse events was 70/972 in the trial group and 48/944 in the control group. Adverse events were as follows: diarrhea (n = 22); skin itching (n = 3); transient aminotransferase mild elevation (n = 3); two cases of rash; nausea (n = 2); and skin allergy (n = 1); all of them were relieved after drug withdrawal, decrement, or symptomatic treatment. Three studies (Peng et al., 2011; Gu et al., 2014; Li, 2014) reported no obvious adverse events. There was only one study (Chen et al., 2010) that reported two cases of SAEs in the treatment group and six SAEs in the control group, but it did not mention the SAEs exactly.

#### Ingredients of CHM Formulae and Frequently Used Herbs

The ingredients of CHM in each RCT are listed in **Supplementary Table 2**. The most frequently used herbs across all formulae were Dahuang (*Radix et Rhizoma Rhei*, rhubarb root and rhizome), Sanqi (*Radix Notoginseng*, Panax notoginseng), Chuanxiong (*Rhizoma Ligustici Chuanxiong*, Ligusticum chuanxiong Hort), Chishao (*Radix Paeoniae Rubra*, Paeonia veitchii Lynch), Shichangpu (*Rhizoma Acori Tatarinowii*, Acorus tatarinowii Schott), Yujin (*Radix Curcumae*, turmeric root tuber), Zhizi (*Fructus Gardeniae*, cape jasmine fruit), Taoren (*Semen Persicae*, peach seed), Shuizhi (*Hirudo*, leech), Honghua (*Flos Carthami*, safflower), Gancao (*Radix Glycyrrhizae*, liquorice root), Danggui (*Radix Angelicae Sinensis*,Angelica sinensis), Niuxi (*Radix Achyranthis Bidentatae*, twotoothed achyranthes root), and Huangqin (*Radix Scutellariae*, baical skullcap root) (**Table 3**).

**Table 3:** Frequently used herbs in included studies.

Chinese name	Pharmaceutical name	Species	Family	Record	Number of studies(%)
**Dahuang**	*Radix et Rhizoma Rhei*	*Rheum palmatum* L.	*Polygonaceae*	–	25(0.56)
**Sanqi**	*Radix Notoginseng*	*Panax notoginseng* (Burkill) F.H.Chen	*Araliaceae*	146751	17(0.38)
**Chuanxiong**	*Rhizoma Ligustici Chuanxiong*	*Ligusticum striatum* DC.	*Apiaceae*	–	16(0.36)
**Chishao**	*Radix Paeoniae Rubra*	*Paeonia lactiflora* Pall.	*Paeoniaceae*	–	15(0.33)
**Shichangpu**	*Rhizoma Acori Tatarinowii*	*Acorus calamus* var. *angustatus* Besser	*Acoraceae*	2306	23(0.51)
**Yujin**	*Radix Curcumae*	*Curcuma aromatica* Salisb. *Curcuma wenyujin* Y.H.Chen & C.Ling	*Zingiberaceae*	235193 235308	11(0.24)
**Zhizi**	*Fructus Gardeniae*	*Gardenia jasminoides* J.Ellis	*Rubiaceae*	88270	10(0.22)
**Taoren**	*Semen Persicae*	*Prunus persica* (L.) Batsch	*Rosaceae*	376	10(0.22)
**Shuizhi**	*Hirudo*	–	*Hirudinidae*	–	9(0.2)
**Honghua**	*Flos Carthami*	*Crocus sativus* L.	*Iridaceae*	327454	9(0.2)
**Gancao**	*Radix Glycyrrhizae*	*Glycyrrhiza uralensis* Fisch.	*Leguminosae*	32406	9(0.2)
**Danggui**	*Radix Angelicae Sinensis*	*Angelica sinensis* (Oliv.) Diels	*Apiaceae*	–	8(0.18)
**Niuxi**	*Radix Achyranthis Bidentatae*	*Achyranthes bidentata* Blume	*Amaranthaceae*	–	8(0.18)
**Huangqin**	*Radix Scutellariae*	*Scutellaria baicalensis* Georgi	*Lamiaceae*	188938	8(0.18)

## Discussion

### Summary of Evidence

This study is an updated systematic review of the efficacy and safety of CHM for ICH. Forty-five low risk of bias RCTs with 4,517 subjects received at least four domains with “yes” according to the Cochrane Risk of Bias tool. The main findings of present systematic review demonstrated that CHM paratherapy can improve dependency, VH, VPE, clinical effective rate, and neurological function deficit; however, it was not significant for improving the mortality rate of ICH patients. In addition, CHM paratherapy had fewer side effects and was generally safe. Although the present study provided supportive evidence of the efficacy and safety of CHM for dependency of ICH, we should treat the results cautiously because the included studies were of high clinical heterogeneity.

### Limitations

First, none of the included studies had been formally registered. Thus, protocols were not available to confirm free of selective reporting (De Angelis et al., 2004). Second, the primary studies existed in some methodological flaws such as distribution concealment and blindness. The selection bias or observer bias may affect the results. Third, only three studies (Huang et al., 2006; Chen et al., 2010; Liu et al., 2012) used CHM placebo in the control group, and thus caution should be given to the interpretation of the positive findings. Fourth, most of the included studies did not carry out formal sample size estimates. Trials with inadequate sample sizes often run the risk of overestimating intervention benefits (Kjaergard et al., 2001). Fifth, the composition of a formula, dosage, administration methods, and duration of CHM treatments varied considerably in the primary RCTs. These clinical heterogeneities may compromise validity of the results of the present study. Sixth, only 18 studies (Jia et al., 2000; Dai et al., 2002; Huang et al., 2006; Chen et al., 2010; Huang et al., 2010; Wang and Wang, 2011; Li et al., 2012; Shen et al., 2012; Zhang et al., 2012; Ming et al., 2013; Wang et al., 2013; Ye, 2014; Li et al., 2015; Li et al., 2016; Liu and Wu, 2016; Liu and Zhang, 2016; Xia et al., 2016; Ma and Zhang, 2017) have more than 3 months’ follow-up. The long prognosis of ICH of CMH treatment at least 6 months needed further clarifying.

### Implications for Practice

The use of CHM in the treatment of ICH has increased in the past decades. The available evidence from the present study is supportive. We summarized the most frequently used 14 herbs: *Radix et Rhizoma Rhei, Radix Notoginseng, Rhizoma Ligustici Chuanxiong, Radix Paeoniae Rubra, Rhizoma Acori Tatarinowii, Radix Curcumae, Fructus Gardeniae, Semen Persicae, Hirudo, FlosCarthami, Radix Glycyrrhizae, Radix Angelicae Sinensis, Radix Achyranthis Bidentatae*, and *Radix Scutellariae*. These selected herbs have far-reaching clinical applications for the treatment based on syndrome differentiation of ICH patients according to the therapeutic functions of herbal medicine. *Radix et Rhizoma Rhei, Radix Notoginseng, Rhizoma Ligustici, Radix Curcumae, Semen Persicae, Hirudo, Flos Carthami, Radix Paeoniae Rubra, Radix Angelicae Sinensis*, and *Radix Achyranthis* have the function of promoting blood circulation for removing blood stasis; *Radix Notoginseng* and *Radix Scutellariae* have the function of hemostasis. Thus, we can deduce that the main pattern of primary ICH is the syndrome of blood stasis blocking brain. In addition, the selected high-frequency herbs can guide the prescribing of clinical treatment of primary ICH and can be used as candidate herbs for RCT.

### Implications for Research

The present study identifies some key areas that contribute to further research. Firstly, the reasons why CHM cannot significantly improve the mortality rate are as follows: A) Sample size: Mortality rate was observed in 14 studies with 106/863 subjects in the treatment group and 179/850 subjects in the control group. There is no statistical difference between the two groups in spite of a presenting trend efficacy. It is difficult to draw conclusions and easy to get false negative results when the sample size is too small (Zhong, 2009). B) Types of participants: The participants’ condition in the included studies was not severe, and some participants with milder conditions were less likely to die. C) Follow-up time: The follow-up time was from 7 days to 1 year, so that we cannot predict the long-term effects of CHM for ICH patients. However, it does not mean that CHM has no significance for improving the mortality rate for ICH patients. The significance of CHM for improving the mortality rate may be undervalued due to the above reasons. Thus, whether CHM can significantly improve the mortality rate of ICH deserves further study. Secondly, it is crucial to improve the methodological quality of RCTs. We recommend that specific guidelines, such as the CONSORT 2010 statement (Schulz et al., 2010), guidelines for RCTs investigating CHM (Flower et al., 2012), and CONSORT Extension for Chinese Herbal Medicine Formulas 2017 (Cheng et al., 2017), should be further used to design and report RCTs for CHM. Thirdly, although the CHM treatment in the included studies was generally safe in ICH patients, the safety of CHM for ICH still needs further confirmation. In addition, the safety of herbal patent injection itself has become a major concern to both national health authorities and the general public (Wang et al., 2010). A standard reporting format for adverse drug reactions (ADR) has been developed (Bian et al., 2010), and we suggest that we should pay close attention to improving the reporting of ADRs of CHM. Fourthly, a longer follow-up period with serial measurements of outcomes is important to determine the genuine effectiveness and long-term effect of ICH. Thus, a longer follow-up period for ICH patients in the design of further clinical trials is needed. Fifthly, disease-syndrome combination is the recognized trend in integrative medicine. Syndrome differentiation is the core of TCM practice, which can establish a TCM treatment principle and enable us to prescribe the herbal formula. Thus, syndrome differentiation is the bridge to prescription corresponding to syndrome (Wang and Xiong, 2012). Thus, when evaluating the efficacy and safety of CHM treatment, the syndrome differentiation of ICH should be considered to further stratify. A precisely tailor-made TCM prescription based on individual differences can help to improve the efficacy of the selected Chinese herbs (Jiang et al., 2012). For instance, one high-quality study published in JAMA (Bensoussan et al., 1998) indicated that using individualized CHM for the treatment of irritable bowel syndrome is more effective than prescribing a common hypnotic prescription. Therefore, based on the syndrome differentiation of patients, we can select the appropriate medicine from the most frequently used 14 herbs in this study in the future clinic treatment, so as to improve the therapeutic effect of CHM for the acute period of ICH. Last but not least, some new sensitive scales can be introduced into future trials, such as the ICH grading scale (Ruiz-Sandoval et al., 2007) and the FUNC score (Rost et al., 2008).

## Conclusion

The present study provided supportive evidence of CHM for improving dependency of ICH and showed general safety; however, there is still lack of evidence for improving mortality rate, and it opens for further study.

## Data Availability Statement

All datasets generated for this study are included in the manuscript/supplementary files.

## Author Contributions

H-LW and HZ contributed as the senior author and the principal investigator of this study, refined the study, and wrote the first draft of the manuscript. G-QZ and QW contributed to the overall design. M-BX and X-LZ identified and reviewed the studies for eligibility. P-QR and T-YJ performed the meta-analysis of the data. All authors read, critically reviewed, and approved the final manuscript.

## Funding

This study was supported by grants from the National Natural Science Foundation of China (81573750/81473491/81173395/H2902), the Young and Middle-Aged University Discipline Leaders of Zhejiang Province, China (2013277), the Zhejiang Provincial Program for the Cultivation of High-level Innovative Health Talents (2015), the National Natural Science Foundation of China (81673627), and Guangzhou Science Technology and Innovation Commission Technology Research Projects (201805010005).

## Conflict of Interest

The authors declare that the research was conducted in the absence of any commercial or financial relationships that could be construed as a potential conflict of interest.
