# Supplementary material for: Efficacy and Safety of Chinese Herbal Medicine for Primary Intracerebral Hemorrhage: A Systematic Review of Randomized Controlled Trials
Source: Front Pharmacol. 2019 Oct 10;10:1139. doi: 10.3389/fphar.2019.01139 (PMC6796400; doi:10.3389/fphar.2019.01139)
Supplement: Supplementary file 1 [file Table_1.pdf]

Supplementary Material

Efficacy and Safety of Chinese Herbal Medicine for Primary Intracerebral Hemorrhage: A Systematic Review of Randomized Controlled Trials

Hui-Lin Wang[1†], Hua Zeng[2†], Meng-Bei Xu[1], Xiao-Li Zhou[1], Pei-Qing Rong[1], Ting-Yu Jin[1], Qi Wang[2*] and Guo-Qing Zheng[1*]

[1]Department of Neurology, The Second Affiliated Hospital and Yuying Children's Hospital of Wenzhou Medical University, Wenzhou, China

[2]Institute of Clinical Pharmacology, Guangzhou University of Chinese Medicine, Guangzhou, China

*** Correspondence:**

Qi Wang, wangqi@gzucm.edu.cn

Guo-Qing Zheng, gq_zheng@sohu.com.

†These authors have contributed equally to this work

SupplementaryTable 1. Characteristics of the Included Studies.

Included trials	Type of disease	Eligibility criteria	Study design	Sample size (male/female), mean age (years)		Mean hematoma volume (Trial/ Control) mL (range)	Onset time of treatment	Interventions(n) drug		Route of administration	Course of treatment	Follow-up	Outcome index	Intergroup differences
				Trial	Control			Trial	Control					
Fan Y et al, 2000	Acute hypertensive cerebral Hemorrhage	CCDDS 1995	RCT	32(19/13), 64.44±12.12	32(17/15), 61.31±11.91	19.22±13.39/ 19.16±9.82	<2d	WCM+Liangxue Tongyu Oral Liquid	WCM	oral administration or nasogastric gavage	4weeks (w)	Not Report (NR)	1.MR (4w)<br>2.Clinical efficacy (4w)<br>3.VH (4w)<br>4.Grade of cerebral edema (4w)	1.P>0.05<br>2.P<0.05<br>3.P>0.05<br>4.P<0.01
Jia YH et al, 2000	Acute hypertensive cerebral hemorrhage	CCDDS 1995	RCT	31(21/10), 62.3±11.6	30(20/10), 62.5±11.0	62.8±10.7/ 58.7±11.4	6~24h	WCM+Stereotactic Drainage+Zhuyu Xiaozhong Mixture	WCM+Stereotactic Drainage treatment	oral administration or nasogastric gavage	1w	6 months(m)	1.Clinical efficacy (6m)<br>2.CCNDS score (6m)<br>3.ADL scale (6m)<br>4.VH (2w)<br>5.MR (6m)	1.P<0.05<br>2.P<0.05<br>3.P<0.05<br>4.P<0.05<br>5.P>0.05
Dia MX et al, 2002	Acute hypertensive cerebral hemorrhage	CCDDS 1995	RCT	40 (26/14), 58.6±10.8	40 (27/13), 58.8±10.5	50.8±11.5/ 50.5±10.8	<2d	WCM+Drilling drainage+Urokinase Dissolution+ Zhuyu Xiaozhong Decoction	WCM+Drilling drainage+Urokinase Dissolution	oral administration or nasogastric gavage	10 days (d)	6m	1.Clinical efficacy (6m)<br>2.CCNDS score (6m)<br>3.average days of the hematoma disappeared<br>4.length of stay<br>5.ADL scale (6m)<br>6.MR	1. P<0.05<br>2.P<0.05<br>3.P<0.05<br>4.P<0.05<br>5.P<0.05<br>6.-
Ma L et al, 2005	Acute intracerebral hemorrhage	CCDDS 1995	RCT	46(35/11), 31±76	46(36/10), 30±78	30.56±7.42/ 29.43±6.85	<2d	WCM+Xuesai Tong Injection	WCM	intravenous injection	14d	NR	1.Clinical efficacy (14d)<br>2.CCNDS score (14d)<br>3.VH (14d)	1.P<0.01<br>2.P<0.01<br>3.P<0.05
Huang PX et al, 2006	Acute hypertensive cerebral hemorrhage	CCDDS 1995	RCT	199(125/74) , 62.9±11.1	205(119/86), 60.9±11.7	22.47±21.09/ 25.89±24.76	<7d	WCM+TCM syndrome differentiation Recipes	WCM+Chinese medicine placebo	Oral or gastric administration or intravenous injection	28d	3m	1.MR<br>2.Clinical efficacy (3m)<br>3.NHISS score (28d,3m)<br>4.BI score (3m)<br>5.Social function questionnaire score	2.P>0.05<br>3.P<0.05<br>4.P<0.05<br>5.P<0.01
Yang JG et al, 2006	Acute intracerebral hemorrhage	CCDDS 1995	RCT	32(20/12)37 ~76(60.8)	30(18/12)38~ 77(59.5)	28.42±11.23/ 27.86±10.84	<2d	WCM+Hematoma Broken Aspiration /extra ventricular drainage+Huatan Tongfu Compound Decoction	WCM+Hematoma Broken Aspiration /extra ventricular drainage	oral administration or nasogastric gavage	14d	NR	1.Clinical efficacy (14d)<br>2.CCNDS score (14d)<br>3.VH (7d,14d)	1.P<0.05<br>2.P<0.01<br>3.P<0.01
Fan YP et al, 2008	Acute intracerebral hemorrhage	CCDDS 1995	single-blind RCT	30(17/13)58 .03±11.89	30(18/12)62.0 0±12.00	12.47±9.60/ 13.03±12.54	<4d	WCM+β-Sodium Aescinate for Injection	WCM	oral administration or nasogastric gavage	14d	NR	1.NIHSS score (7d)<br>2.severity of the surrounding edema (7d)<br>3.serum NO 、TNF-α 、IL-6 、MMP-9 (7d)	1.P>0.05<br>2.P>0.05<br>3.P>0.05
Sun JH et al,	Acute	CCDDS	RCT	45(28/17)	44(25/197)	30-49	7~72h	WCM+Minimally Invasive	WCM+MIS treatment	oral administration or	21d	30d	1.NIHSS score (14d,30d)	1.P<0.05

2008	intracerebral hemorrhage	1995		58.36±16.54	59.62±15.93			Surgery (MIS) treatment +Salvia miltiorrhiza Injection		nasogastric gavage			2.VPE (7d,14d,21d)<br>3.MR	2.P<0.05<br>3.P>0.05
Liao H et al, 2010	Acute intracerebral hemorrhage	CCDDS 1995	RCT	30(21/9)50.3±7.5(45-68)	30(17/13)52.9±6.9(43-70)	18.3±7.7/17.2±8.9	<1d	WCM+Tian Huang granule	WCM	Unreported	28d	NR	1.intracranial pressure (2d,3d,4d,5d,6d,7d)<br>2.serum MMP-9 standard (3d,5d,7d)<br>3.Hydrocephalic volume (3d,7d,14d)<br>4.VH (3d,7d,14d)<br>5.NIHSS score (3d,7d,14d,28d)	1.P<0.05 (7d)<br>2.P<0.05 (5d)<br>P<0.01 (7d)<br>3.P<0.05 (7d)<br>P<0.01 (14d)<br>4.P<0.05 (7d)<br>P<0.01 (14d)<br>5.P<0.05 (14d)<br>P<0.01 (28d)
Chen SH et al, 2010	Acute intracerebral hemorrhage	CCDDS 1995	Multicenter, double-blind, placebo RCT	108,>45	105,>45	>5	<3d	WCM+Zhongfeng Xingnao Oral Liquid	WCM+Zhongfeng Xingnao Oral Liquid placebo	oral administration or nasogastric gavage	30d	90d	1.MR<br>2.mRS score≥4<br>3.NIHSSscore≤1 (90d)<br>4.GOS score (90d)<br>4.mRSscore≤2 (90d)<br>5.BI score (90d)	1.P<0.05<br>2.P<0.05<br>3.P<0.05<br>4.P<0.05<br>5.P<0.05
Huang JL et al, 2010	Acute intracerebral hemorrhage	CCDDS 1995	RCT	45(29/16)<br>61.38	45(29/16)<br>61.38	>20	6~12h	WCM+surgery+Dahuang Zhilong Decoction	WCM+surgery	oral administration or nasogastric gavage	3m	90d	1.The number of people with sobriety and sobriety (7d,14d,30d)<br>2.CCNDS score (30d,90d)<br>3.MR (90d)<br>4.Clinical efficacy (90d)	1.P<0.01(7d, 30d)<br>2.P<0.05 (90d)<br>3.-<br>4.P<0.01
Ming SP et al, 2010	Acute intracerebral hemorrhage	CCDDS 1995	RCT	46(29/17)<br>56±10	45(29/16)<br>62±9	29.2±9.9/<br>29.4±7.7	<2d	WCM+Xingnao Jing Injection	WCM	intravenous injection	14d	NR	1.VH (7d,21d)<br>2.VPE (7d,21d)<br>3.ESS score (7d,21d)	1.P<0.01 (21d)<br>2.P<0.01 (21d)<br>3.P<0.05 (7d,21d)
Chen Y, 2011	Acute hypertensive intracerebral basal ganglia hemorrhage	CCDDS 1995	RCT	30(22/8)<br>63.2	30(23/7)<br>64.1	<30	<2d	WCM+Xingnao Kaiqiao Decoction	WCM	oral administration or nasogastric gavage	14d	NR	1.Clinical efficacy (14d)<br>2.VH (14d)<br>3.CCNDS score (14d)	1.P<0.05<br>2.P<0.05<br>3.P<0.05
Li WZ et al, 2011	Acute hypertensive cerebral hemorrhage	CCDDS 1995	RCT	88(56/32)<br>67.9±13.48	85(51/34)<br>66.1±15.20	25.65±9.72/<br>24.23±10.65	<1d	WCM+Xingshen Kaiqiao Decoction Concentrate	WCM	oral administration or nasogastric gavage	28d	NR	1.VH (7d,14d,21d,28d)<br>2.Conscious time	1.P<0.05 (14d,21d,28d)<br>2.-
Peng GJ et al, 2011	Acute intracerebral Hemorrhage	1996 Chinese Cerebrovascular Disease Diagnosis Standard	RCT	40(27/13)<br>51±9.42	40(25/15)<br>50±10.1	18.4±7.3/<br>20.1±5.9	<1d	WCM+Tianlong Tongjing Recipe	WCM	oral administration	28d	NR	1.Improved Edinburgh Scandinavian score method (28d)<br>2.VH (14d,28d)<br>3.VPE (14d,28d)	1.P<0.05<br>2.P>0.05<br>3.P<0.05

Wang ZP et al, 2011	Acute hypertensive cerebral hemorrhage	CCDDS 1995	RCT	90(55/35)<br>60.78±5.69	89(53/36)<br>61.24±5.68	24.36±4.25/<br>24.75±4.11	<3d	WCM+Tongqiao Huoxue Decoction (acute stage) and Buyang Huanwu Decoction (at recovery phase)	WCM	oral administration or nasogastric gavage	14-90d	90d	1.Survival rate (90d)<br>2.Cumulative survival ladder (90d)<br>3.Number of changes in hematoma volume (14d)<br>4.CCNDS score (90d)<br>5.ADL scale (90d)	1.P>0.05<br>2.-<br>3.P<0.05<br>4.P<0.05<br>5.P<0.05
Li YY et al, 2012	Acute intracerebral Hemorrhage	CCDDS 1995	RCT	46(26/19)<br>57.82±11.86	45(30/15)<br>55.08±12.43	25.82±8.93/<br>23.86±8.01	<1d	WCM+Apoplexy Recipe	WCM	oral administration or nasogastric gavage	30d	3m	1.CCNDS score (30d,90d)<br>2.Clinical efficacy (30d,90d)<br>3. BI score (30d,90d)<br>4.ADL scale (90d)	1.P<0.05<br>2.P<0.05<br>3.P<0.05<br>4.P<0.05
Liu JR et al, 2012	Acute hypertensive cerebral hemorrhage	CCDDS 1995	RCT	44(21/23)<br>62.3±4.6	46(22/24)<br>62.1±5.8	48.44±10.30<br>/<br>49.21±10.10	<1d	WCM+micro-damage puncture+Tongfu Xiere Compound Decoction	WCM+micro-damage puncture+Tongfu Xiere Compound placebo Decoction	oral administration or nasogastric gavageor rectal administration	4d	48d	1.MR<br>2.ADL scale (18d,48d)<br>3.CCNDS score (18d,48d)<br>4.Clinical efficacy (18d,48d)<br>5.plasma ET 、NO standard (24h,6d,12d,18d)	1.-<br>2.P<0.05<br>3.P<0.05<br>4.P<0.01<br>5.P<0.01
Shen LQ et al, 2012	Acute hypertensive intracerebral basilar nucleus hemorrhage	CCDDS 1995	RCT	35(23/12)<br>64.91±8.47	35(24/11)<br>65.24±7.82	16.23±5.94/<br>15.41±5.62	<3d	WCM+Xuesai Tong Injection	WCM	intravenous injection	14d	3m	1.VH (14d)<br>2.Degree of cerebral edema (14d)<br>3.plasma D dimer, CRP standard (1d,3d,7d,14d)<br>4.NIHSS score (14d,3m)	1.P<0.05<br>2.P<0.05<br>3.P<0.05<br>4.P<0.05
Zhang SQ et al, 2012	Acute intracerebral Hemorrhage	CCDDS 1995	RCT	45(25/20)<br>55.70±12.16	45(26/19)<br>56.10±11.45	28.20±5.30/<br>28.85±3.25	1~ 3d	WCM+Bushen Huoxue Huatan Decoction	WCM	Unreported	8w	3m	1.VH (7d,14d,28d)<br>2.VPE (7d,14d,28d)<br>3.NIHSS score (7d,14d,28d,60d,90d)<br>4.Clinical efficacy (90d)	1.P<0.05<br>2.P<0.05<br>3.P<0.05<br>4.P<0.05
Ming SP et al, 2013	Acute hypertensive cerebral hemorrhage	CCDDS 1995	RCT	40(25/15)<br>58 ±8.6	40(26/14)<br>61±2.9	19.2±7.8/<br>18.2±9.8	<3d	WCM+Raw rhubarb powder	WCM	oral administration or nasogastric gavage	10d	3m	1.ESS score (14d,30d,90d)	1.P<0.05
Wang YQ et al, 2013	Acute hypertensive cerebral hemorrhage	CCDDS 1995	RCT	29(28/8)<br>63.28±10.19	41(30/11)<br>63.37±9.84	28.77±5.68/<br>28.77±5.68	<3d	WCM+Xuefu Zhuyu Decoction Oral Liquid	WCM	oral administration or nasogastric gavage	4-12w	12m	1.VH (2w,1m)<br>2.CCNDS score (2w,1m,3m,1year)	1.P<0.05 (1m)<br>2.P<0.05 (3m,1year)
Bi XL et al, 2014	Acute intracerebral Hemorrhage	CCDDS 1995	RCT	44(27/17)<br>63.5±10.7	42(26/16)<br>62.3±9.6	13.54±2.55/<br>13.42±2.46	<7d	WCM+Xueshuan Tong Injection	WCM	intravenous injection	2w	NR	1.VH (2w)<br>2.NIHSS score degree (2w)	1.P<0.05<br>2. P<0.05

Gu HJ et al,<br>2014	Acute hypertensive cerebral hemorrhage	CCDDS<br>1995	RCT	55(26/29)<br>64.5±14.5	55(28/27)<br>66.4±16.4	-	<1d	WCM+Xingnao Jing Injection	WCM	intravenous injection	7d	NR	1.GCS score (1d,3d,7d)<br>2.NIHSS score (1d,3d,7d)<br>3.serumhs-CRP Content (1d,3d,7d)<br>4.serumNSE mass concentration (1d,3d,7d)	1.P<0.05 (7d)<br>2. P<0.05 (7d)<br>3. P<0.05 (3d,7d)<br>4. P<0.01 (7d)
Guo LJ et al,<br>2014	Acute hypertensive intracerebral basal ganglia hemorrhage	CCDDS<br>1995	RCT	60(37/23)<br>64.54±9.82	60(35/25)<br>61.92±10.65	19.46±4.04/<br>20.32±6.58	<3d	WCM+Shuizhi Huoxue Sanzhong Oral Liquid	WCM	Unreported	14d	NR	1.VH (14d)<br>2.Clinical efficacy (14d)	1.P<0.05<br>2.P<0.05
Li P<br>2014	Acute hypertensive cerebral hemorrhage	CCDDS<br>1995	RCT	43(26/17)<br>58.5±12.3	43(28/15)<br>57.8±11.5	19.55±5.86/<br>18.38±6.23	<2d	WCM+Salvia miltiorrhiza lyophilized powder needle	WCM	intravenous injection	14d	NR	1.Clinical efficacy (14d)<br>2.VH (1w,2w)<br>3.VPE (1w,2w)<br>4.NIHSS score (1w,2w)	1.P<0.05<br>2.P<0.05 (2w)<br>3.P<0.05 (2w)<br>4.P<0.05 (2w)
Ye R<br>2014	Acute hypertensive cerebral hemorrhage	CCDDS<br>1995	RCT	48(31/17)<br>70.2±6.5	42(29/13)<br>69.5±7.2	22.8±5.7/<br>23.1±6.6	<1d	WCM+MIS treatment+TCM syndrome differentiation Recipes	WCM+MIS	Unreported	3m	3m	1.Clinical efficacy (3m)<br>2.VH (7d,21d)<br>3.VPE (7d,21d)<br>4.Stroke score (15d,1m,3m)	1.P<0.05<br>2.P<0.05<br>3.P<0.05<br>4.P<0.05
Guan JJ<br>et al,<br>2015	Acute hypertensive cerebral hemorrhage	CCDDS<br>1995	RCT	42(24/18)<br>64.5±9.3	42(26/16)<br>63.7±8.5	37.5±11.9/<br>35.7±10.5	<2d	WCM+Ginkgo leaf extract and dipyridamole Injection	WCM	intravenous injection	14d	NR	1.Clinical efficacy (75%) (14d)<br>2.NIHSS score (14d)<br>3.GCS score (14d)<br>4.Fugl-Meyer Motor function evaluation scale (14d)<br>5.serumNSE, complement C3, C4, hs-CRP standard (14d)	1.P<0.05<br>2.P<0.01<br>3.P<0.01<br>4.P<0.01<br>5.P<0.01
Li K<br>et al,<br>2015	Acute hypertensive cerebral hemorrhage	CCDDS<br>1995	RCT	62(33/29)<br>69.63±11.35	60(32/28)<br>71.34±12.21	20-40	<3d	WCM+Zhongfeng Xingnao Oral Liquid	WCM	oral administration or nasogastric gavage	28d	3m	1.Clinical efficacy (3m)<br>2.ESS score (3m)	1.P<0.05<br>2.P<0.05
Luo ZJ et al,<br>2015	Acute hypertensive cerebral hemorrhage	CCDDS<br>1995	RCT	58(28/30)<br>67.4±12.6	62(32/30)<br>66.2±13.8	25.58±9.41/<br>25,24±9.52	<2d	WCM+Xuesai Tong Injection	WCM	intravenous injection	14d	17d	1.VH (1d,4d,10d,17d)<br>2.NIHSS score (1d,4d,10d,17d)	1.P<0.05 (10d,17d)<br>2.P<0.05 (10d)<br>P<001 (17d)
Peng WX<br>et al,<br>2015	Acute hypertensive cerebral hemorrhage	CGCPT<br>2007	RCT	36(23/13)<br>55.1±7.2	35(22/13)<br>54.5±7.3	20.6±4.1/<br>20.9±4.1	3d	WCM+Liuwei Naoxue An Decoction	WCM	oral administration or nasogastric gavage	28d	NR	1.Clinical efficacy (28d)<br>2.VH (28d)<br>3.CCNDS score (28d)	1.P<0.01<br>2.P<0.01<br>3.P<0.01
Shen Y et al,<br>2015	Acute hypertensive cerebral	CCDDS<br>1995	RCT	33(21/12),<br>63.89±11.24	32(20/12),<br>64.71±10.05	26.73±5.84/<br>27.85±6.17	<3d	WCM+Xuefu Zhuyu Decoction	WCM	NR	4w	NR	1.Clinical efficacy (4w)<br>2.VH (4w)<br>3.CCNDS score (4w)	1.P<0.05<br>2.P<0.05<br>3.P<0.05

	hemorrhage												4.Hemorheological indicators (total blood viscosity, plasma viscosity, blood cell specific volume, fibrinogen, platelet adhesion rate, thrombin time) (4w)	4.P<0.05
Li JY et al, 2016	Acute intracerebral Hemorrhage	CCDDS 1995	Multicenter, opened-RCT	114(71/43), 59.27±12.10	114(70/44), 62.06±10.75	12.86±11.44/ 13.02±11.31	<5d	WCM+TCM syndrome differentiation Recipes +Xingnao Jing Injection (14d) +Naoxue Shu Oral Liquid (60d, after 21 days' hospitalization)	WCM	oral administration or intravenous injection	3m	3m	1.mRS score (21d,3m)<br>2.NIHSS score (7d,14d,21d,3m)<br>3.Clinical efficacy (3m)<br>4.VH (7d)<br>5.patient reported outcomes (PRO) (7d,14d,21d,3m)	1.P<0.01 (3m)<br>2.P<0.05 (7d ,21d,3m)<br>3.P>0.05<br>4.P>0.05<br>5.P<0.05 (21d,3m)
Guo J et al, 2016	Acute intracerebral Hemorrhage	CCDDS 1995	RCT	41(23/18), 52.6	32(18/14), 56.7	30-65,45.2 /30-63,45.6	<3d	WCM+MIS+Xingnao Guanchang Decoction	WCM+MIS	rectal administration	7d	NR	1.MR (7d)<br>2.Clinical efficacy (7d)<br>3.GCS score (24h,48h)<br>4.average length of stay	1.-<br>2.P<0.05<br>3.P<0.05<br>4.P<0.05
Jiang SS et al, 2016	Acute hypertensive cerebral hemorrhage	CCDDS 1995	RCT	34(19/15), 54.78±5.03	34(18/16), 54.12±5.89	≤50	<5d	WCM+Huoxue Ditan Decoction	WCM	oral administration or nasogastric gavage	3w	NR	1.Clinical efficacy (21d)<br>2.NIHSS score (7d,14d,21d)<br>3.VH (21d)	1.P<0.05<br>2.P<0.05<br>3.P<0.05
Liu SW et al, 2016	Acute hypertensive cerebral hemorrhage	CGCPT 2007	RCT	41(25/16), 61.50±10.30	41(26/15), 61.30±10.70	-	<3d	WCM+Xingnaojing Injection+TCM syndrome differentiation Recipe Granule	WCM	oral administration or intravenous injection	3w	3m	1.Clinical efficacy (3m)<br>2.NIHSS score (3m)	1.P<0.05<br>2.P<0.05
Liu YC et al, 2016	Acute intracerebral Hemorrhage	CCDDS 1995	RCT	40(21/19), 60.90±5.60	38(22/16), 61.50±5.80	60-150/40-120	<3d	WCM+Qingre Huayu Decoction	WCM	oral administration or gastric administration	4w	12m	1.NIHSS score (4w)<br>2.serum NSE standard (4w)<br>3.clinic prognosis (1 year)<br>4.MR	1.P<0.05<br>2.P<0.05<br>3.P<0.05<br>4.-
Long YJ et al, 2016	Acute hypertensive intracerebral basal ganglia hemorrhage	CGCPT 2007	RCT	42(22/20), 61.78±7.53	38(22/16), 61.50±5.80	33.19±5.18/ 32.83±5.18	<2d	WCM+MIS+Annao Pingchong Tablet	WCM+MIS	oral administration or naso gastric gavage	2w	NR	1.CCNDS score (1w,2w)<br>2.GCS score (1w,2w)<br>3.VPE (1w,2w)<br>4.serum AQP4 standard (1w,2w)<br>5.Clinical efficacy (2w)	1.P<0.01<br>2.P<0.01<br>3.P<0.05<br>4.P<0.01<br>5.P<0.01
Shang QM et al, 2016	Acute intracerebral Hemorrhage	CCDDS 1995	RCT	40(28/12), 60.2	40(26/14), 59.6	-	<3d	WCM+Xingnao Jing Injection+Chinese Medicine Decoction	WCM	oral administration or nasogastric gavage or gastric administration or intravenous injection	3w	1w	1.NIHSS score (2d,7d,14d,30d)<br>2.Clinical efficacy (30d)	1.P<0.05 (14d,30d)<br>2.P<0.05

Xia ZY et al, 2016	Acute hypertensive cerebral hemorrhage	CCDDS 1995	Multicenter, randomized-double-blind RCT	26(16/10), 59.2	25(18/7), 64.1	-	<6h	WCM+Poxue Zhuyu Decoction Granule	WCM+Not Poxue Zhuyu herb Granule+Chinese herb medicine placebo Granule	oral administration or nasogastric gavage	3w	3m	1.24h Swelling rate<br>2.NIHSS score (3m)<br>3.mRS score (3m)	1.P>0.05<br>2.P>0.05<br>3.P>0.05
Zhou W et al, 2016	Acute intracerebral Hemorrhage	CCDDS 1995	RCT	40(26/14), 50.31±7.11	40(28/12), 50.66±7.28	21.16±6.36/ 20.31±6.02	<3d	WCM+Buyang Huanwu Decotion	WCM	oral administration	2w	1m	1.MR (2w)<br>2.GCS score (2w)<br>3.NIHSS score (2w)<br>4.inflammatory factor (hs-CRP, VEGF, Hpa) (2w)<br>5.Overall clinical efficiency (2w)	1.P>0.05<br>2.P<0.05<br>3.P<0.05<br>4.P<0.05<br>5.P<0.05
Lei XN et al, 2017	Acute intracerebral Hemorrhage	CGCPT 2007	RCT	50(32/18), 62.9±8.1	50(29/21), 60.7±9.50	17.52±6.26/ 15.16±6.85	<10d	WCM+Qingre Huotan TongfuDecoction	WCM	oral administration or nasogastric gavage	28d	NR	1.MR<br>2.VH (7d,28d)<br>3.NIHSS score (7d,28d)<br>4.Clinical efficacy (28d)<br>5.Stroke specific quality of life scale (28d)	1.-<br>2.P<0.05 (28d)<br>3.P<0.05 (28d)<br>4.P>0.05<br>5.P<0.05
Ma HB et al, 2017	Acute hypertensive cerebral hemorrhage	CCDDS 1995	RCT	59(32/27), 68.9±11.3	59(36/23), 65.9±9.80	37.6±11.3/ 35.2±10.7	<1d	WCM+SalviaLigustrazine Injection	WCM	intravenous injection	28d	3m	1.GCS score (14d)<br>2.NIHSS score (14d)<br>3.serum CRP, endothelin 1 (14d)<br>4.Grade of cerebral edema (14d)<br>5.Prognosis of Glasgow score (3m)	1.P<0.05<br>2.P<0.05<br>3.P>0.05<br>4.P<0.05<br>5.P<0.05
Sun MF et al, 2017	Acute hypertensive cerebral hemorrhage	1998 Chinese Cerebrovascular Disease Diagnosis Standard	RCT	34(16/18), 57.67±3.72	34(19/15), 59.34±4.12	28.69±11.46/ 28.71±11.34	<1d	WCM+Xingnao Kaiqiao Zhuyu Decoction	WCM	oral administration	21d	NR	1.Clinical efficacy (21d)<br>2.NIHSS score (21d)<br>3.VH (21d)	1.P<0.05<br>2.P<0.05<br>3.P<0.05
Zhang F et al, 2017	Acute hypertensive cerebral hemorrhage	CCDDS 1995	RCT	54(37/17), 65.2±7.1	54(31/23), 63.4±6.9	30-100	<1d	WCM+MIS+Huoxue Sanyu Xingnao Decoction	WCM+MIS	NR	14d	NR	1.Neurological deficit score (14d)<br>2.Clinical efficacy (14d)<br>3.MR<br>4.serum MMP-9 standard (2d,7d,14d)	1.P<0.05<br>2.P<0.05<br>3.-<br>4.P<0.05 (7d,14d)

CCDDS 1995, Chinese Cerebrovascular Disease Diagnosis Standard, 1995; CGCPT 2007, Chinese Guidelines for Cerebral hemorrhage Prevention and Treatment 2007; WCM, western conventional medicine; MIS, minimally invasive surgery; RCT, randomized controlled trials; NIHSS, National Institutes of Health Stroke Scale; CCNDS, Chinese Clinical Neurological Deficit Scale; GCS, Glasgow Coma Scale; ESS, Europe Stroke score; BI, Barthel index; mRS, modified Rankin standard; ADL, activity of daily living; MR, mortality rate; VH, volume of hematoma; VPE: volume of perihematomal edema; NR, not reported;-, not reported; d, day; w, week; m, month.
