# Supplementary material for: Efficacy and Safety of Chinese Herbal Medicine for Primary Intracerebral Hemorrhage: A Systematic Review of Randomized Controlled Trials
Source: Front Pharmacol. 2019 Oct 10;10:1139. doi: 10.3389/fphar.2019.01139 (PMC6796400; doi:10.3389/fphar.2019.01139)
Supplement: Supplementary file 2 [file Table_2.pdf]

Supplementary Material

Efficacy and Safety of Chinese Herbal Medicine for Primary Intracerebral Hemorrhage: A Systematic Review of Randomized Controlled Trials

Hui-Lin Wang[1†], Hua Zeng[2†], Meng-Bei Xu[1], Xiao-Li Zhou[1], Pei-Qing Rong[1], Ting-Yu Jin[1], Qi Wang[2*] and Guo-Qing Zheng[1*]

[1]Department of Neurology, The Second Affiliated Hospital and Yuying Children's Hospital of Wenzhou Medical University, Wenzhou, China

[2]Institute of Clinical Pharmacology, Guangzhou University of Chinese Medicine, Guangzhou, China

*** Correspondence:**

Qi Wang, wangqi@gzucm.edu.cn

Guo-Qing Zheng, gq_zheng@sohu.com.

†These authors have contributedequally to this work

Supplementary Table 2. Ingredients of CHM formulae.

Study (years)	Prescription	Ingredients of herb prescription	Usage of prescription	Preparations	Quality control
Fan Y et al, 2000	Liangxue Tongyu Oral Liquid	*Radix et Rhizoma Rhei* 10g,*Cornu Bubali* 30g, *Radix rehmanniae recens* 15g, *Semen Persicae* 10g	60 ml/bid (2.18g/1ml)	Oral Liquid	Produced by Chinese medicine academy of Nanjing university of Chinese medicine
Jia YH et al, 2000	Zhuyu Xiaozhong Mixture	*Hirudo, Rhizoma Ligustici Chuanxiong, Radix Paeoniae Rubra, Semen Persicae, Flos Carthami, Fructus Jujubae*	33 ml/tid (0.44g/1ml)	Oral Liquid	Produced by Chengdu hospital ofintegrated traditional Chinese and western medicine
Dia MX et al, 2002	Zhuyu Xiaozhong Decoction	*Hirudo, Rhizoma Ligustici Chuanxiong, Radix Paeoniae Rubra, Semen Persicae, Flos Carthami, Radix et Rhizoma Rhei, Radix Scutellariae, Fructus Jujubae*	1/2 dose/bid	Decoction	Unreported
Ma L et al, 2005	XuesaiTong Injection	*Panax Notoginseng Saponins*	5ml×2+NS 250 ml/qd (0.05g/1ml)	Injection	Produced by Kunming pharmaceutical group Co., Ltd.

Huang PX et al, 2006	Angong Niuhuang Pill	*Artificial Calculus Bovis, Cornu Bubali, Moschus, Pernulo, Cinnabaris, Realgar, Rhizoma Coptidis, Radix Scutellariae, Fructus Gardeniae, Radix Curcumae, Borneolum Syntheticum*	1/2#/bid, 3 d (3g/1#) oral or gastric administration	Tablet	Produced by Beijing Tongren Tang pharmaceutical factory, Traditional Chinese patented medicine WY: Z11020193, batchnumber:1010063
	Annao Pill	*Rhizoma Coptidis, Radix Scutellariae, Pernulo, Fructus Gardeniae, Radix Curcumae, Haematitum, Realgar, Cinnabaris, Gypsum Fibrosum, Borneolum Syntheticum, Cornu Bubali, ArtificialCalculus Bovis*	1#/bid, 28d or 25d (3g/1#) oral or gastric administration	Tablet	Produced by Heilongjiang lushen pharmaceutical group Co., Ltd.
	Tongfu Xingshen Capsule	*Folium Sennae, Rhizoma Polygoni Cuspidati, ArtificialCalculus Bovis, Concretio Silicea Bambusae, Fructus Trichosanthis*	4#/tid, 10d (0.5g/1#) oral or gastric administration	Capsule	Produced by the traditional Chinesemedicine hospital of Guangdong province
	Qingkai Ling Injection	*Cholic Acid, Concha Margaritifera, Hyodeoxycholicacid, Fructus Gardeniae, Cornu Bubali, Radix Isatidis, Radix Scutellariae, Flos Lonicerae*	40 ml + NS 500 ml/qd, 21d (1g/1ml) intravenous injection	Injection	Produced by the traditional Chinese medicine of Beijinguniversity' s pharmaceutical factory
	Agreement PrescriptionNo.1	*Artificia lCalculus Bovis, Cornu Bubali, Radix Gentianae*	1#/qd, 28d (8.9g/1#) oral or gastric	Granule	Produced by Jiangyin pharmaceutical factory in Jiangsu Province

		administration		
Suhe Xiang Pill	*Styrax, Benzoinum, Borneolum Syntheticum, Cornu Bubali, Moschus, Lignum Santali Albi, Lignum Aquilariae Resinatum, Flos Caryophylli, Rhizoma Cyperi, Radix Aucklandiae, Olibanum, Fructus Piperis Longi, Rhizoma Atractylodis Macrocephalae, Fructus Chebulae, Cinnabaris*	1/2#/bid, 3d oral or gastric administration	Tablet	Produced by Beijing Tongren Tang pharmaceutical factory
Huatuo Zaizao pill	*Radix Angelicae Sinensis, Rhizoma Ligustici Chuanxiong, Flos Carthami, Rhizoma Arisaematis, Semen Strychni, Fructus Evodiae, Borneolum Syntheticum*	6g/bid, 28d or 25d oral or gastric administration	Tablet	Produced by Guangzhou Qixingpharmaceutical Co., Ltd.
Tongfu Xingshen Capsule	*Folium Sennae, Rhizoma Polygoni Cuspidati, Artificial Calculus Bovis, Concretio Silicea Bambusae, Semen trichosanthis*	4#/tid, 10d (0.5g/1#) oral or gastric administration	Capsule	Produced by the traditional Chinese medicine hospital of Guangdong province
Danshen Injection	*Radix Salviae Miltiorrhizae, Lignum Dalbergiae Odoriferae*	20ml+5% Glucose Injection 500ml/qd, 21d (1g/1ml) intravenous injection	Injection	Produced by Yaan Sanjiu Pharmaceutical Co., Ltd.

	Agreement Prescription No.2	*Rhizoma Gastrodiae, Rhizoma Ligustici Chuanxiong, Rhizoma Arisaematis*	1#/qd, 28d (10.6 g/1#) Oral or gastric administration	Granule	Produced by Jiangyin pharmaceutical factory in Jiangsu Province
Yang JG et al, 2006	Huatan Tongfu Decoction	*Radix et Rhizoma Rhei* 10g, *Rhizoma Arisaematis Cum Bile* 10g, *Natrii Sulfas* 10g, *Fructus Gardeniae* 10g, *Rhizoma Acori Tatarinowii* 10g, *Radix Polygalae* 10g, *Caulis Bambusae in Taenia* 10g, *Fructus Aurantii Immaturus* 10g, *Fructus Trichosanthis* 20g, *Radix Scutellariae* 15g, *Pericarpium Citri Reticulatae* 12g, *Poria* 20g	1/2dose/bid, 3-7d	Decoction	Unreported
Fan YP et al, 2008	β-aescine sodium Injection	*aescine*	0.1-0.4mg/kg or 5-10mg+10% Glucose Injection 250ml or NS 250ml/qd, 14d	Injection	Unreported
Sun JH et al, 2008	Salvia miltiorrhiza Injection	*Radix Salviae Miltiorrhizae*	20ml+5% Glucose Injection 250ml/qd, 21d	Injection	Unreported
Liao H et	Tianhuang	*Radix Notoginseng, Radix et Rhizoma Rhei,* and *Borneolum*	10g/bid, 28d	Decoction	Produced by the Chinese

al, 2010	Granule	*Syntheticum* in the ratio of 1:4:0.1			drugpharmaceutical department of the Forth Central hospital of Tianjin city
Chen SH et al, 2010	Zhongfeng Xingnao Oral Liquid	*Radix Ginseng Rubra, Radix Notoginseng, Radix et Rhizoma Rhei*	10ml×12/d, 30d	Oral Liquid	Produced by the affiliated hospital of Chengdu university of traditional Chinese medicine
Huang JL et al, 2010	Dahuang Zhidan Decoction	*Radix et Rhizoma Rhei* 15g, *Hirudo* 10g, *Radix Salviae Miltiorrhizae* 30g, *Radix Notoginseng* 3g, *Radix Scutellariae* 20g, *Lumbricus* 20g, *Rhizoma Acori Tatarinowii* 20g, *Rhizoma Arisaematis Cum Bile* 30g	1/2dose/bid	Decoction	Unreported
Ming SP et al, 2010	XingnaoJing Injection	*Moschus, Borneolum Syntheticum, Fructus Gardeniae, Radix Curcumae*	20ml/+NS 250ml/qd, 14d	Injection	Produced by Wuxi Jimin Kexin Shanhe pharmaceutical Co., Ltd.; Traditional Chinese patented medicine WY:Z32020564
Chen Y, 2011	Xingnao Kaiqiao Decoction	*Radix Panacis Quinquefolii* 6g, *Radix Notoginseng* 15g, *Rhizoma Arisaematis Cum Bile* 15g, *Moschus* 0.15g, *Radix et Rhizoma Rhei* 6g, *Radix Salviae Miltiorrhizae* 10g, *Radix*	1/2dose/bid, 14d	Decoction	Produced by Chen Y

		Curcumae 10g			
Li WZ et al, 2011	Xingshen Kaiqiao Oral Liquid	*Radix Rehmanniae Preparata* 25g, *Radix Ophiopogonis* 10g, *Herba Dendrobii* 15g, *Fructus Corni* 25g, *Herba Cistanches* 20g, *Rhizoma Acori Tatarinowii* 10g, *Radix Polygalae* 10g, *Rhizoma Pinelliae* 10g, *Radix Notoginseng* 10g, *Radix et Rhizoma Rhei* 6g, *Semen Persicae* 10g, *Rhizoma Ligustici Chuanxiong* 15g, *Radix Scutellariae* 10g, *Pollen Typhae* 15g, *Herba Leonuri* 20g, *Rhizoma Curcumae* 10g, *Poria* 20g	125-250ml/qd or bid, 28d	Decoction	Produced by department of neurosurgery in the Tianshui hospital of integrated traditional Chinese and western medicine
Peng GJ et al, 2011	Tianlong Tongjing Decoction	*Rhizoma Gastrodiae* 10g, *Lumbricus* 15g, *Ramulus Uncariae Cum Uncis* 15g, *Radix Paeoniae Rubra* 10g, *Radix Gentianae Macrophyllae* 15g, *Ramulus Mori* 30g, *Ramulus Cinnamomi* 15g, *Caulis Spatholobi*30g, *Herba Lycopi* 15g, *Poria* 15g, *Radix Glycyrrhizae* 6g	150ml/bid, 28d	Decoction	Provided by the pharmacy of Traditional Medicine of the affiliated hospital of Hebei university
Wang ZP	Tongqiao Huoxue	*Radix Paeoniae Rubra* 12g, *Rhizoma Ligustici Chuanxiong* 12g, *Semen Persicae*20g, *Flos Carthami* 20g, *Fructus Jujubae* 7g,	1dose/qd, 14-90 d	Decoction	Unreported

et al, 2011	Decoction (in theacute stage)	*shallot* 3g, *Rhizoma Zingiberis Recens* 9g, *Moschus* 0.15g			
	Buyang Huanwu Decoction (in the convalescence)	*Radix Astragali seu Hedysari* 60g, *Radix Paeoniae Rubra* 15g, *Rhizoma Ligustici Chuanxiong* 15g, *Radix Angelicae Sinensis* 20g, *Lumbricus* 15g, *Semen Persicae* 12g, *Flos Carthami* 15g	1dose/qd, 14-90 d	Decoction	Unreported
Li YY et al, 2012	Apoplexy Recipe	*Radix Angelicae Sinensis, Rhizoma Ligustici Chuanxiong, Radix Paeoniae Rubra,Radix Astragali seu Hedysari, Hirudo, Rhizoma et Radix Notopterygii, Radix Glycyrrhizae*	15g/12h, 30d	Decoction	Produced by the GansuprovincialhospitalofTCM; Traditional Chinese patented medicine WY:Gansu Health (1997)-098-04
Liu JR et al, 2012	Tongfu Xiere Compound Decoction	*Radix et Rhizoma Rhei* 15g, *Radix Paeoniae Rubra* 15g, *Radix Cyathulae* 15g, *Natrii Sulfas* 10g, *Rhizoma Coptidis* 10g, *Cortex Phellodendri* 10g, *Cortex Moutan Radicis* 10g, *Semen Persicae* 10g, *Flos Carthami* 10g, *Lumbricus* 12g, *Radix Angelicae Sinensis* 6g, *Radix Glycyrrhizae* 6g	80ml/qd, oral administration or nasogastric gavage; 150ml/qd, rectal administration, 4d	Decoction	Produced by the first affiliated hospital of Guangzhou university of traditional Chinese medicine
Shen LQ	Xuesai Tong	*Panax Notoginseng Saponins*	400mg+NS	Injection	Produced by Kunming Xingzhong

et al, 2012	Injection		250ml/qd, 14d		pharmaceutical factory; batchnumber:072381
Zhang SQ et al, 2012	Bushen Huoxue Huatan Decoction	*Radix Polygoni Multiflori Preparata* 20g*, Fructus Corni* 15g*, Rhizoma Dioscoreae* 15g,*Radix Ophiopogonis* 15g*, Herba Dendrobii* 15g*, Fructus Schisandrae Chinensis* 5g*, Herba Cistanches* 15g*, Rhizoma Acori Tatarinowii* 10g*, Radix Curcumae* 10g*, Radix Notoginseng* 6g*, Radix et Rhizoma Rhei* 5-10g*, Poria* 30g*, Radix Angelicae Sinensis* 30g*, Rhizoma Ligustici Chuanxiong* 30g*, Radix Salviae Miltiorrhizae* 30g*, Herba Leonuri* 30g*, Hirudo* 10g*, Radix Glycyrrhizae* 5g	1/2dose (200ml)/bid, 56d	Decoction	Unreported
Ming SP et al, 2013	Raw rhubarb powder	*Radix et Rhizoma Rhei* 3-15g	3-15g/bid, 10d	Decoction	Unreported
Wang YQ et al, 2013	Xuefu Zhuyu Decoction	*Semen Persicae, Flos Carthami, Radix Angelicae Sinensis, Rhizoma Ligustici Chuanxiong, Radix Rehmanniae Recens, Radix Paeoniae Rubra, Radix Achyranthis Bidentatae, Radix Bupleuri, Fructus Aurantii, Radix Platycodonis, Radix Glycyrrhizae*	10 ml/tid, 28d	Oral Liquid	Produced by the Jilin Aodongpharmaceutical Co., Ltd.; Traditional Chinese patented medicine WY:Z10950063

Bi XL et al, 2014	Xueshuan Tong Injection	*Panax Notoginseng Saponins*	300mg+NS 250ml/qd, 14d	Injection	Unreported
Gu HJ et al, 2014	Xingnao Jing Injection	*Moschus, Borneolum Syntheticum, Fructus Gardeniae, Radix Curcumae*	30ml+5% Glucose Injection 250ml or NS 250ml/qd, 7d	Injection	Produced by the Wuxi Jimin Kexin Shanhepharmaceutical Co., Ltd.; Traditional Chinese patented medicineWY:Z32020564
Guo LJ et al, 2014	Shuizhi Huoxue Sanzhong Oral Liquid	*Hirudo, Radix et Rhizoma Rhei, Eupolyphaga Seu Steleophaga, Radix Notoginseng, Radix Salviae Miltiorrhizae, Herba Leonuri, Scorpio, Concretio Silicea Bambusae*	10ml/bid	Oral Liquid	Produced by Yutian county hospital of traditional Chinese medicine; Traditional Chinese patented medicine WY:Z20110091; batchnumber:20130105
Li P 2014	Danshen Freeze dried Powder Injection	*Radix Salviae Miltiorrhizae*	0.4g+5% Glucose Injection 250ml or NS 250ml/qd, 14d	Injection	Produced by the Harbin pharmaceutical group Co., Ltd.
Ye R	Lingjiao Gouteng	*Cornu Saigae Tataricae, Bulbus Fritillariae Cirrhosae, Radix Rehmanniae Recens, Ramulus Uncariae Cum Uncis, Rhizoma*	1dose	Decoction	Unreported

2014	Decoction	*Gastrodiae, Flos Chrysanthemi, Poria cum Radix Pini, Radix Paeoniae Rubra, Radix Achyranthis Bidentatae, Radix Glycyrrhizae*	(250ml)/bid, 90d		
	Daotan Chengqi Decoction	*Fructus Aurantii Immaturus, Rhizoma Arisaematis Cum Bile, Fructus Trichosanthis, Rhizoma Pinelliae Preparatum, Pericarpium Citri Reticulatae, Cortex Magnoliae Officinalis, Natrii Sulfas, Radix et Rhizoma Rhei, Rhizoma Atractylodis Macrocephalae, Fructus Gardeniae, Radix Glycyrrhizae*	1dose (250ml)/bid, 90d	Decoction	Unreported
	Ditan Decoction	*Poria, Fructus Aurantii Immaturus, Rhizoma Arisaematis Cum Bile, Rhizoma Pinelliae Preparatum, Exocarpium Citri Rubrum, Rhizoma Acori Tatarinowii, Fructus Trichosanthis, Radix Curcumae, Caulis Bambusae in Taenia, Radix Glycyrrhizae, Rhizoma Zingiberis Recens*	1dose (250ml)/bid, 90d	Decoction	Unreported
	Shenfu Decoction	*Radix Ginseng, Radix Aconiti Lateralis Preparata, Rhizoma Zingiberis Recens, Fructus Jujubae*	1dose (250ml)/bid, 90d	Decoction	Unreported
	Huatan Quyu Huoluo Decoction	*Rhizoma Acori Tatarinowii, Rhizoma Arisaematis Cum Bile, Radix Paeoniae Rubra, Rhizoma Ligustici Chuanxiong, Radix Achyranthis Bidentatae, Radix Curcumae*	1dose (250ml)/bid, 90d	Decoction	Unreported
	Yougui	*Radix Rehmanniae Preparata, Rhizoma Dioscoreae, Radix*	1dose	Decoction	Unreported

	Decoction and Zhigancao Decoction	*Aconiti Lateralis Preparata, Cortex Cinnamomi, Colla Corii Asini, Radix Glycyrrhizae, Radix Codonopsis, Fructus Corni, Radix Ophiopogonis, Fructus Cannabis, Fructus Lycii, Cortex Eucommiae*	(250ml)/bid, 90d		
	Dading Fengzhu Decoction	*Carapax et Plastrum Testudinis, Carapax Trionycis, Concha Ostreae, Radix Rehmanniae Recens, Radix Paeoniae Alba, Radix Ophiopogonis, Colla Corii Asini, Fructus Schisandrae Chinensis, Radix Glycyrrhizae*	1dose(250ml)/bid, 90d	Decoction	Unreported
	Zhengan Xifeng Decoction	*Haematitum, Os Draconis, Concha Ostreae, Carapax et Plastrum Testudinis, Radix Achyranthis Bidentatae, Radix Paeoniae Alba, Radix Scrophulariae, Radix Asparagi, Fructus Meliae Toosendan, Herba Artemisiae Scopariae, Fructus Hordei Germinatus*	1dose (250ml)/bid, 90d	Decoction	Unreported
Guan JJ et al, 2015	Ginkgo Leaf Extract and Dipyridamole Injection	*Total Flavone of Ginkgo, Dipyridamole*	5ml×4+5%-10% Glucose Injection 500ml or NS 500ml/bid, 14d	Injection	Produced by Guizhou Yibaipharmaceutical Co., Ltd.;Traditional Chinese patented medicine WY:H52020032
Li K et al,	Zhongfeng Xingnao Oral	*Radix Ginseng Rubra, Radix Notoginseng, Radix et Rhizoma Rhei*	25ml/qid, 28d	Oral Liquid	Produced by the affiliated hospital of Chengdu university of traditional Chinese medicine; Traditional Chinese patented

2015	Liquid				medicine WY:Z20070623
Luo ZJ et al, 2015	Xuesai Tong Injection	*Panax Notoginseng Saponins*	0.4g+NS 250ml/qd, 14d	Injection	Unreported
Peng WX et al, 2015	Liuwei Naoxue An Decoction	*Radix Cyathulae, Hirudo, Radix et Rhizoma Rhei, Cornu Bubali, Radix Salviae Miltiorrhizae, Radix Notoginseng*	100 ml/bid, 28d	Decoction	Produced by Hangzhou hospital of traditional Chinese medicine
Shen Y et al, 2015	Xuefu Zhuyu Decoction	*Radix Angelicae Sinensis* 15g, *Rhizoma Ligustici Chuanxiong* 10g, *Radix Rehmanniae Recens* 15g, *Radix Paeoniae Rubra* 15g, *Semen Persicae* 15g, *Flos Carthami* 10g, *Radix Achyranthis Bidentatae* 10g, *Radix Bupleuri* 10g, *Fructus Aurantii* 10g, *Radix Platycodonis* 10g, *Radix Glycyrrhizae* 6g	1dose/d, 28d	Decoction	Unreported
Li JY et al,	a formula to treat blood stasis accompanied by	*Radix Notoginseng* 10g, *Pollen Typhae* 9g, *Radix et Rhizoma Rhei* 9g,*Radix Scutellariae* 20g,*Rhizoma Curcumae Longae* 10g, *Poria* 20g, *Fructus Gardeniae* 10g,*Fructus Trichosanthis* 20g	Unreported	Decoction	Unreported

2016	stagnation of heat toxin				
	a formula to treat blood stasis accompanied by internal stirring of liver wind	*Radix Notoginseng* 10g, *Pollen Typhae* 9g, *Haliotis asinina Linnaeus* 15g, *Radix Paeoniae Alba*12g, *Rhizoma Curcumae* 10g, *Poria* 20g, *Ramulus Uncariae Cum Uncis* 30g, *Rhizoma Gastrodiae* 12g	Unreported	Decoction	Unreported
	a formula to treat blood stasis accompanied by phlegm blocking channels and collaterals	*Radix Notoginseng* 10g, *Pollen Typhae* 9g, *Rhizoma Pinelliae* 10g, *Radix Trichosanthis* 20g, *Rhizoma Curcumae* 10g, *Poria* 20g, *Radix et Rhizoma Rhei* 3g	Unreported	Decoction	Unreported
	Xingnaojing Injection	*Moschus, Borneolum Syntheticum, Fructus Gardeniae, Radix Curcumae*	20ml+NS 250ml/qd, 14d<br>intravenous injection	Injection	Produced by the Wuxi Jimin Kexin Shanhe Pharmaceutical Co., Ltd.; Traditional Chinese patented medicine WY:Z32020564<br>, batch number:110305
	Naoxueshu	*Radix Astragali seu Hedysari, Hirudo, Rhizoma Acori Tatarinowii, Radix Achyranthis Bidentatae, Cortex Moutan*	10ml/tid, 60d	Oral	Produced by the Shandong Wohua Pharmaceutical Co., Ltd.; Traditional

	Oral Liquid	*Radicis, Radix et Rhizoma Rhei, Rhizoma Ligustici Chuanxiong*	oral	Liquid	Chinese patented medicine WY:Z20070059; batch number:101205
Guo J et al, 2016	Xingnao Guanchang Decoction	*Rhizoma Arisaematis Cum Bile* 15g, *Rhizoma Acori Tatarinowii* 20g, *Fructus Trichosanthis* 20g, *Radix et Rhizoma Rhei* 12g, *Fructus Gardeniae* 10g,*Radix Curcumae* 12g, *Cortex Magnoliae Officinalis* 15g, *Borneolum Syntheticum* 3g	200ml/qd, 7d	Decoction	Unreported
Jiang SS et al, 2016	Huoxue Ditan Decoction	*Radix Salviae Miltiorrhiza* 30g, *Fructus Trichosanthis* 15g, *Eupolyphaga Seu Steleophaga* 15g, *Rhizoma Arisaematis Cum Bile* 10g, *Fructus Aurantii Immaturus* 10g, *Radix et Rhizoma Rhei* 10g, *Rhizoma Acori Tatarinowii* 15g, *Radix Curcumae* 10g, *Rhizoma Ligustici Chuanxiong* 10g, *Cortex Moutan Radicis* 10g, *Concretio Silicea Bambusae* 10g, *Exocarpium Citri Rubrum* 10g	1dose/d (300ml for 2-3 times), 21d	Decoction	Unreported
Liu SW et al, 2016	Xingnaojing Injection	*Moschus, Borneolum Syntheticum, Fructus Gardeniae, Radix Curcumae*	20ml+NS 250ml/qd, 14d, intravenous injection	Injection	Produced by Wuxi Jimin Kexin Shanhe pharmaceutical Co., Ltd.; Traditional Chinese patented medicine WY:Z32020564

	a formula to treat blood stasis accompanied by internal stirring of liver wind	*Radix Notoginseng* 10g, *Rhizoma Gastrodiae* 12g, *Pollen Typhae* 9g, *Rhizoma Curcumae* 10g, *Ramulus Uncariae Cum Uncis* 15g, *Concha Haliotidis* 30g, *Radix Paeoniae Rubra* 12g, *Poria* 20g	2times/d, oral administration, 21d	Granule	Produced by Beijing Kang Rentang pharmaceutical Co., Ltd.
	a formula to treat blood stasis accompanied by phlegm blocking channels and collaterals	*Radix Notoginseng* 10g, *Pollen Typhae* 9g, *Rhizoma Curcumae* 10g, *Radix et Rhizoma Rhei* 3g, *Rhizoma Pinelliae* 10g, *Fructus Trichosanthis* 30g, *Poria* 20g	2times/d, oral administration, 21d	Granule	Produced by Beijing Kang Rentang pharmaceutical Co., Ltd.
	a formula to treat stagnation of heat toxin	*Radix Notoginseng* 6g, *Pollen Typhae* 9g, *Rhizoma Curcumae* 10g, *Radix et Rhizoma Rhei* 3g, *Radix Astragali seu Hedysari* 10g, *Fructus Gardeniae* 10g, *Fructus Trichosanthis* 20g, *Poria* 20g	2times/d, oral administration, 21d	Granule	Produced by Beijing Kang Rentang pharmaceutical Co., Ltd.
Liu YC et al, 2016	Qingre Huayu Decoction	*Cornu Bubali* 25g, *Radix Notoginseng* 15g, *Radix Paeoniae Rubra* 12g, *Lumbricus* 6g, *Radix et Rhizoma Rhei* 12g, *Concha Ostreae* 25g, *Concretio Silicea Bambusae* 12g, *Rhizoma Ligustici Chuanxiong* 10g, *Rhizoma Acori Tatarinowii* 12g, *Radix Glycyrrhizae* 6g	1/2dose/bid, 28d	Decoction	Unreported

Long YJ et al, 2016	Annao Pingchong Tablet	*Os Draconis* 30g, *Concha Ostreae* 30g, *Radix AchyranthisBidentatae* 15g, *Fructus Gardeniae* 12g, *Radix Scutellariae* 12g, *Ramulus Uncariae Cum Uncis* 12g, *Radix Aucklandiae* 12g, *Rhizoma Alismatis* 12g, *Radix et Rhizoma Rhei* 9g, *Periostracum Cicadae* 6g, *Radix Bupleuri* 6g, *Radix Glycyrrhizae* 6g	4#/tid, 14d	Tablet	Produced by the first affiliated hospital of Hunan university of traditional Chinese medicine; batch number:20140217
Shang QM et al, 2016	Xingnaojing Injection	*Moschus, Borneolum Syntheticum, Fructus Gardeniae, Radix Curcumae*	20ml+5% Glucose Injection 250ml or NS 250ml/qd, 7d, intravenous injection	Injection	Unreported
	Chinese Medicine Decoction	*Radix Astragali seu Hedysari* 60g, *Radix Codonopsis* 30g, *Radix Paeoniae Rubra* 30g, *Radix Salviae Miltiorrhizae* 30g, *Lumbricus* 3g, *Rhizoma Ligustici Chuanxiong* 6g, *Flos Carthami* 6g, *Rhizoma Atractylodis Macrocephalae* 10g, *Poria* 10g	1/3dose/tid, 15d, oral administration or nasogastric gavage	Decoction	Unreported

Xia ZY et al, 2016	Poxue Zhuyu Decoction Granule	*Hirudo, tabanus, Radix et Rhizoma Rhei, Pollen Typhae, Radix Trichosanthis, Radix Notoginseng,Chinemys reevesii, Rhizoma Acori Tatarinowii*	1dose/bid, 10d	Granule	Produced by Traditional Chinese Medicine Hospital of Guangdong Province
Zhou W et al, 2016	Buyang Huanwu Decotion	*Radix Astragali seu Hedysari* 20g, *Radix Angelicae Sinensis* 15g, *Rhizoma Ligustici Chuanxiong* 15g, *Semen Persicae* 10g, *Flos Cartham i*10g, *Radix Paeoniae Rubra* 10g, *Lumbricus*10g. (In the early acute stage, added *Rhizoma Gastrodiae* 10g, *Ramulus Uncariae Cum Uncis* 10g, *Radix et Rhizoma Rhei* 5g; in the acute peak period, added*Rhizoma Gastrodiae* 10g, *Ramulus Uncariae Cum Uncis* 10g, *Poria* 10g, *Radix Salviae Miltiorrhizae* 10g; in the convalescence, added *Radix Salviae Miltiorrhizae* 20g, *Rhizoma Arisaematis Cum Bile* 15g, *Poria* 10g)	1/3dose/tid, 14d	Decoction	Unreported
Lei XN et al, 2017	Qingre Huotan Tongfu Decoction	*Radix Rehmanniae Recens* 20g, *Carapax et Plastrum Testudinis* 15g, *Cortex Moutan Radicis* 15g, *Flos Chrysanthemi* 10g, *Fructus Gardeniae* 10g, *Radix Scutellariae* 10g, *Spica Prunellae* 10g, *Radix Paeoniae Alba* 10g, *Rhizoma Pinelliae Preparatum* 10g, *Poria* 10g,*Caulis Bambusae in Taenia* 10g, *Fructus Aurantii Immaturus* 10g,*Cortex Magnoliae Officinalis* 10g,*Rhizoma Acori Tatarinowii* 10g, *Rhizoma Arisaematis Cum Bile* 6g, *Radix et Rhizoma Rhei* 5g	Bid, after meal	Decoction	Produced by Yinchuan hospital of traditional Chinese medicine

Ma HB et al, 2017	Salvia Ligustrazine Injection	*Salvianic acid A, ligustrazine hydrochloride*	10ml+NS 250ml/qd, 28d intravenous injection	Injection	Produced by the Guizhou Baite pharmaceutical Co., Ltd.; Traditional Chinese patented medicine WY:H52020959
Sun MF et al, 2017	Xingnao Kaiqiao Zhuyu Decoction	*Radix Notoginseng* 3g, *Herba Menthae* 10g, *Rhizoma Acori Tatarinowii* 10g, *Spica Prunellae* 10g, *Radix Paeoniae Alba* 15g, *Ramulus Uncariae Cum Uncis* 15g, *Concha Haliotidis* 15g, *Radix Angelicae Sinensis* 6g, *Radix Salviae Miltiorrhizae* 10g, *Radix Astragali seu Hedysari* 10g, *Radix Achyranthis Bidentatae* 15g, *Herba Leonuri* 15g, *Radix et Rhizoma Rhei* 12g	1/2dose/bid, 21d	Decoction	Unreported
Zhang F et al, 2017	Huoxue Sanyu Xingnao Decoction	*Radix Astragali seu Hedysari* 60g, *Radix Angelicae Sinensis* 15g, *Rhizoma Ligustici Chuanxiong* 15g, *Semen Persicae* 12g, *Flos Carthami* 20g, *Radix Paeoniae Rubra* 20g, *Rhizoma Gastrodiae* 15g, *Ramulus Uncariae Cum Uncis* 15g, *Lumbricus* 10g, *Radix et Rhizoma Rhei* 12g, *Fructus Aurantii Immaturus* 10g, *Radix Notoginseng* 3g	1/2dose/bid, 14d	Decoction	Unreported

Note: Since valid taxonomic names were not provided, the composition of the preparations used remains unclear. *d, day; bid: bis in die; qd: quaque die; tid: ter in die; #: tablet; Co., Ltd: Company Limited; NS: 0.9% Sodium Chloride Injection.*
